# Comparative Phospho- and Acetyl Proteomics Analysis of Posttranslational Modifications Regulating Intestine Regeneration in Sea Cucumbers

**DOI:** 10.3389/fphys.2018.00836

**Published:** 2018-07-03

**Authors:** Lina Sun, Chenggang Lin, Xiaoni Li, Lili Xing, Da Huo, Jingchun Sun, Libin Zhang, Hongsheng Yang

**Affiliations:** ^1^CAS Key Laboratory of Marine Ecology and Environmental Sciences, Institute of Oceanology, Chinese Academy of Sciences, Qingdao, China; ^2^Laboratory for Marine Ecology and Environmental Science, Qingdao National Laboratory for Marine Science and Technology, Qingdao, China

**Keywords:** sea cucumber, intestine regeneration, phosphoproteomics, acetyl proteomics, posttranslational modification

## Abstract

Sea cucumbers exposed to stressful circumstances eviscerate most internal organs, and then regenerate them rapidly under favorable environments. Reversible protein phosphorylation and acetylation are major modifications regulating protein function. Herein, for the first time, we perform quantitative phospho- and acetyl proteomics analyses of intestine regeneration in a sea cucumber species *Apostichopus japonicus*. We identified 1,862 phosphorylation sites in 1,169 proteins, and 712 acetylation sites in 470 proteins. Of the 147 and 251 proteins differentially modified by phosphorylation and acetylation, respectively, most were related to cytoskeleton biogenesis, protein synthesis and modification, signal recognition and transduction, energy production and conversion, or substance transport and metabolism. Phosphorylation appears to play a more important role in signal recognition and transduction than acetylation, while acetylation is of greater importance in posttranslational modification, protein turnover, chaperones; energy production and conversion; amino acid and lipid transport and metabolism. These results expanded our understanding of the regulatory mechanisms of posttranslational modifications in intestine regeneration of sea cucumbers after evisceration.

## Introduction

The ability to regenerate viscera is the most dramatic characteristic of sea cucumbers. Sea cucumbers exposed to stressful circumstances eviscerate most internal organs including the digestive tube, the haemal system, and the respiratory trees; and they can restore the lost organs in 20~100 days when placed in a favorable environment, and regain full functions (García-Arrarás and Greenberg, 2001). This phenomenon has aroused the interest of many researchers who are dedicated to studying mechanisms responsible for the regulation of this phenomenal regenerative capacity (Vickery et al., 2001; Carnevali, 2006; Candia-Carnevali et al., 2009; García-Arrarás and Dolmatov, 2010).

Over the past two decades, a number of researchers have investigated many mechanisms in sea cucumbers, including those related to morphological changes, cell differentiation, proliferation and migration, extracellular matrix remodeling, nerve regrowth, and gene regulatory mechanisms (García-Arrarás et al., 1998, 1999; García-Arrarás and Greenberg, 2001; Mashanov et al., 2011, 2013; Sun et al., 2013b; Miao et al., 2017). However, the mechanisms controlling regeneration have not been fully understood due to the complexity of this process and the interferences of multiple genes during transcription, post-transcriptional regulation, translation, and posttranslational modification at multiple levels (Zhao et al., 2016). Thanks to the rapid development of high-throughput sequencing technologies, considerable mRNA and microRNA expression profiles related to regeneration of sea cucumbers have been reported, which investigated the regenerative mechanism at the genome-wide scale (Ortiz-Pineda et al., 2009; Sun et al., 2011, 2013a, 2017a,b; Mashanov et al., 2014). These findings provide comprehensive insight into the underlying mechanisms of regeneration, and a roadmap for screening and functional analysis of key candidate genes. However, despite of this progress, few proteome studies related to sea cucumbers regeneration have been reported (Sun et al., 2017c).

In our previous study, we used isobaric tag for relative and absolute quantitation (iTRAQ) technology to probe proteomic changes during intestine regeneration in *Apostichopus japonicus* (Sun et al., 2017c). However, due to the complexity of regeneration, it's far from revealing the mechanism only at protein expression levels. The posttranslational modification (PTM) regulation of regeneration such as phosphorylation and acetylation need to be investigated. Protein phosphorylation is universally employed for temporary modulation of protein function, serving to alternatively induce or abolish enzyme activity, and facilitate or disrupt protein interactions (Huttlin et al., 2010). Protein acetylation is also an important regulatory modification that regulates diverse functions including DNA recognition, protein-protein interactions, and protein stability (Kouzarides, 2000). Both modifications play important and well-characterized roles in many biological processes related to regeneration including signal transduction, nervous system development (Cohen et al., 2011), hepatic regeneration (Revuelta-Cervantes et al., 2011), intestinal regeneration (Cai et al., 2010), development of male germ cells (Pang and Rennert, 2013), and regulation of embryonic stem cell transcription (Kim J. et al., 2010). Proteomic-based approaches provide powerful tools for investigation of complex biological processes (Franco et al., 2013). Moreover, quantitative phospho- and acetyl proteomics analyses have been successfully applied in many studies including the mechanism of aestivation in sea cucumbers (Chen et al., 2016), regulation of resistant rice (Li et al., 2015), regulation of development in the murine brain (Goswami et al., 2012), regulation of mouse cardiomyopathy (Kuzmanov et al., 2016), and pathological processes in type 2 diabetes (Du et al., 2015).

**Figure 1:**
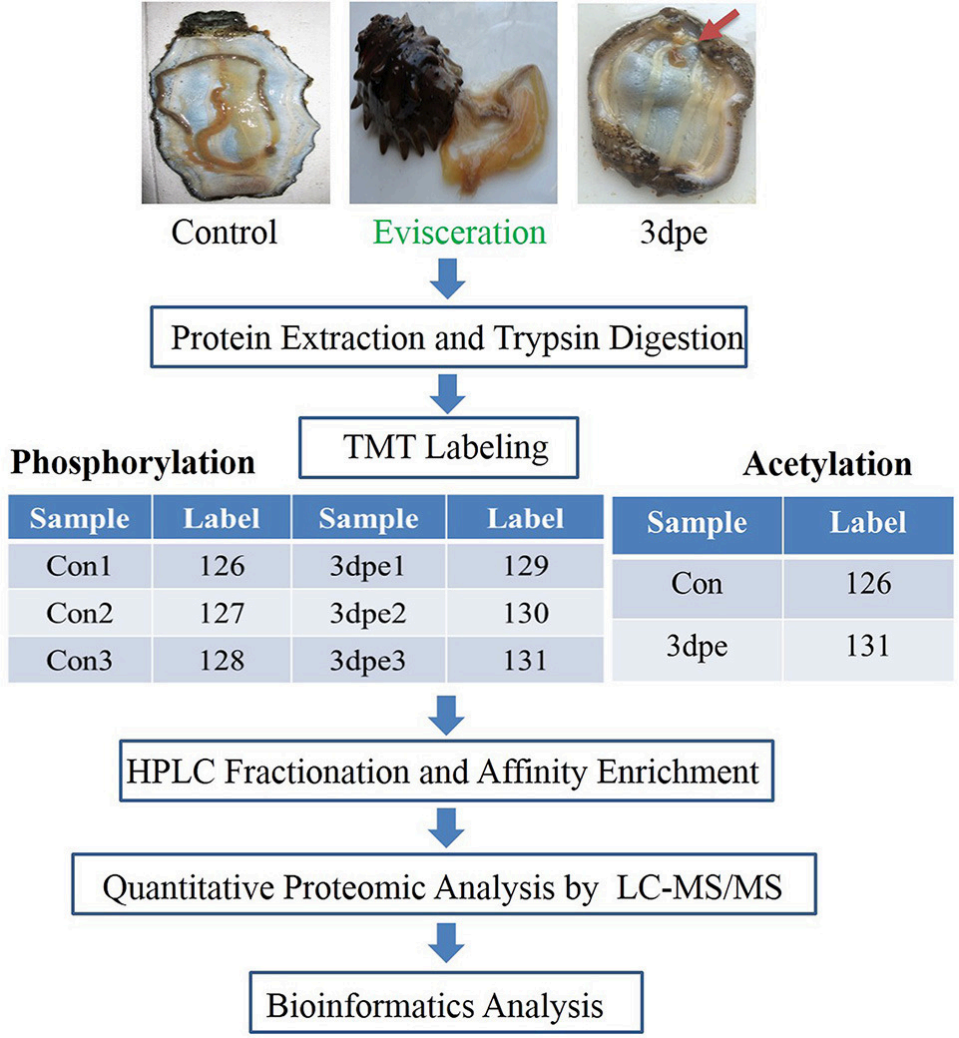
Outline of the experiment design for phosphorylation and acetylation modification study. Control, the normal intestine in the sea cucumber. 3 dpe, the regenerative intestine at 3 days post evisceration.

Herein, we employed tandem mas tag (TMT) labeling and immobilized metal affinity chromatography (IMAC) enrichment followed by tandem high resolution liquid chromatography-mass spectrometry (LC-MS/MS) quantitative phospho- and acetyl proteomics analyses of regenerative intestine at 3 days post evisceration (dpe) in *A. japonicus* (Figure 1). Intestine regeneration can be divided into five different stages: wound healing (0–3 dpe), blastema formation (3–7 dpe), lumen formation (7–14 dpe), intestine differentiation (14–21 dpe), and growth (21 dpe-) (García-Arrarás et al., 1998; Ortiz-Pineda et al., 2009). At 3 dpe, regeneration enters the blastema formation stage that includes the most crucial regulatory processes during intestine regeneration involving cell migration, dedifferentiation, and transdifferentiation, during which many genes are differentially expressed (García-Arrarás et al., 1998; Sun et al., 2013a). At this stage, intestine regeneration was found to be regulated by genes related to cytoskeletal changes, protein synthesis, signal recognition and transduction, energy production and conversion, and substance transport and metabolism (Sun et al., 2017c). This is the first analysis of PTM-related mechanisms regulating intestine regeneration in sea cucumbers, and the first to describe the importance of acetylation in sea cucumbers. The results provided new insight into the posttranslational regulatory mechanisms responsible for regeneration in sea cucumbers.

## Materials and methods

### Animals and samples

Adult *A. japonicus* (100 ± 10 g) were collected from the coast of Qingdao, Shandong Province, and acclimated in an aquarium containing seawater at ~15°C. Control sea cucumbers were fed once a day. For the regeneration experiment, sea cucumbers were induced to eviscerate all viscera, including intestine and respiratory trees by injecting ~2 mL 0.35 M KCl into the coelom (García-Arrarás and Greenberg, 2001; Suárez-Castillo, 2004; Rojas-Cartagena et al., 2007). For the phosphorylation study, regenerative intestine from 12 individuals (three biological replicates × four sea cucumbers per biological replicate) at 3 days post-evisceration (dpe) were included in the experiment group (three biological replicates: 3dpe1, 3dpe2, and 3dpe3). Normal intestine from 12 non-eviscerated sea cucumbers (three biological replicates × four sea cucumbers per biological replicate) were collected to serve as the control group (three biological replicates: Con1, Con2, and Con3). For the acetylation study, regenerative intestine from five individuals at 3 dpe were used for experiment group 3 dpe, and normal intestine from five non-eviscerated sea cucumbers served as controls. Normal and regenerative intestines were immediately frozen in liquid nitrogen and stored at −80°C.

### Protein extraction and trypsin digestion

Dissected intestines were first ground with liquid nitrogen, and the cell powder was transferred to a 5 mL centrifuge tube and sonicated three times on ice using a high intensity ultrasonic processor (Scientz) in lysis buffer (8 M urea, 2 mM EDTA, 10 mM DTT, and 2% phosphatase inhibitor cocktail V). The remaining debris was removed by centrifugation at 20,000 × g for 10 min at 4°C. Finally, protein was precipitated with cold 15% trichloroacetic acid (TCA) for 2 h at −20°C. After centrifugation at 4°C for 10 min, the supernatant was discarded, and the remaining precipitate was washed with cold acetone three times. Protein was redissolved in buffer (8 M urea, 100 mM NH_4_HCO_3_, pH 8.0) and the protein concentration was determined with a 2-D Quant kit (GE Healthcare, Piscataway NJ) according to the manufacturer's instructions.

For the phosphorylation study, the protein solution was reduced with 10 mM dithiothreitol (DTT) for 1 h at 37°C and alkylated with 20 mM iodoacetamide (IAA) for 45 min at room temperature in darkness. For the acetylation study, the protein solution was reduced with 5 mM DTT for 30 min at 56°C and alkylated with 11 mM IAA for 15 min at room temperature in darkness. For trypsin digestion, the method was the same for both studies. Briefly, protein samples were diluted by adding 100 mM TEAB to less than 2 M urea, and trypsin was added at a 1:50 trypsin:protein mass ratio for the first digestion overnight, followed by a second 1:100 trypsin:protein digestion for 4 h.

### TMT labeling, HPLC fractionation, and affinity enrichment

After trypsin digestion, peptides were desalted using a Strata X C_18_ SPE column (Phenomenex) and vacuum-dried. The peptides were reconstituted in 0.5 M TEAB and processed according to the manufacturer's protocol supplied with the 6-plex TMT kit (Figure 1). Briefly, one unit of TMT reagent (defined as the amount of reagent required to label 1 mg of protein) was thawed and reconstituted in acetonitrile (ACN). Peptide mixtures were then incubated for 2 h at room temperature, pooled, desalted, and dried by vacuum centrifugation.

For the phosphorylation study, the sample was fractionated by high pH reversed-phase HPLC with an Agilent 300Extend C_18_ column (5 μm particles, 4.6 mm inside diameter, 250 mm length). Peptides were first separated into 80 fractions with a gradient of 2–60% ACN in 10 mM ammonium bicarbonate (pH 10) over 80 min. The peptide mixtures were first incubated with an IMAC microsphere suspension and shaken gently. IMAC microspheres enriched with phosphopeptides were collected by centrifugation, and the supernatant was removed. For the phosphorylation study, to remove non-specifically adsorbed peptides, IMAC microspheres were sequentially washed with 50% ACN/6% TFA and 30% ACN/0.1% TFA. To elute the enriched phosphopeptides from the IMAC microspheres, elution buffer containing 10% NH4OH was added and gently shaken. The supernatant containing phosphopeptides was collected and lyophilised for LC-MS/MS analysis.

For the acetylation study, the sample was fractionated by high pH reversed-phase HPLC using a Thermo Betasil C_18_ column (5 μm particles, 4.6 mm ID, 250 mm length). The peptides were first separated into 60 fractions with a gradient of 8–32% ACN (pH 9.0) over 60 min. The peptides were then combined into eight fractions and dried by vacuum centrifuging. The peptides were dissolved in IP buffer solution (100 mM NaCl, 1 mM EDTA, 50 mM TRIS-HCl, and 0.5% NP-40, pH 8.0) and the supernatant was transferred to acetylated resin (PTM104 from PTM Bio Company). Following incubation with shaking at 4°C overnight, and washing sequentially with 50% ACN/6% TFA and 30% ACN/0.1% TFA, salt was removed from the acetylated peptides using C_18_ ZipTips prior to LC-MS/MS analysis.

### Quantitative proteomic analysis by LC-MS/MS

#### Phosphorylation analysis

Peptides were dissolved in solvent A consisting of 0.1% formic acid (FA) in 2% ACN and directly loaded onto a reversed-phase pre-column (Acclaim PepMap 100, Thermo Fisher Scientific). Peptide separation was performed using a reversed-phase analytical column (Acclaim PepMap RSLC, Thermo Fisher Scientific) with a linear gradient of 4–22% solvent B (0.1% FA in 98% ACN) for 50 min, 22–35% solvent B for 12 min, 35–80% solvent B for 4 min, and holding at 80% for the last 4 min, all at a constant flow rate of 400 nL/min, on an EASY-nLC 1000 UPLC system. The resulting peptides were analyzed by a Q Exactive Plus Hybrid Quadrupole-Orbitrap mass spectrometer (Thermo Fisher Scientific).

Peptides were subjected to an NSI source followed by tandem mass spectrometry (MS/MS) in a Q Exactive Plus (Thermo Fisher Scientific) coupled online to the UPLC. Intact peptides were detected in the Orbitrap at a resolution of 70,000. Peptides were selected for MS/MS using an NCE setting of 28, 30, and ion fragments were detected in the Orbitrap at a resolution of 17,500. A data-dependent procedure that alternated between one MS scan followed by 20 MS/MS scans was applied for the top 20 precursor ions above a threshold ion count of 5.0E3 in the MS survey scan with 15.0 s dynamic exclusion. The electrospray voltage applied was 2.0 kV. Automatic gain control (AGC) was used to prevent overfilling of the orbitrap, and 5E4 ions were accumulated for generation of MS/MS spectra. For MS scans, the m/z scan range was 350–1,800. The fixed first mass was set as 100 m/z.

#### Acetylation analysis

Tryptic peptides were dissolved in 0.1% FA (solvent A), directly loaded onto a homemade reversed-phase analytical column (15 cm length, 75 μm inside diameter). The gradient comprised an increase from 6 to 23% solvent B (0.1% FA in 98% ACN) over 26 min, 23–35% over 8 min, an increase to 80% over 3 min, then holding at 80% for the last 3 min, all at a constant flow rate of 400 nL/min on an EASY-nLC 1000 UPLC system. The peptides were subjected to an NSI source followed by MS/MS analysis in an Orbitrap Fusion Tribrid (Thermo Fisher Scientific) coupled online to the UPLC. The intact peptides were detected in the Orbitrap at a resolution of 60,000. The peptides were selected for MS/MS using an NCE setting of 35, and ion fragments were detected in the Orbitrap at a resolution of 15,000. A data-dependent procedure that alternated between one MS scan followed by 20 MS/MS scans was applied for the top 20 precursor ions above a threshold intensity greater than 5E3 in the MS survey scan with 15.0 s dynamic exclusion. The electrospray voltage applied was 2.0 kV. AGC was used to prevent overfilling of the Orbitrap, and 5E4 ions were accumulated for generation of MS/MS spectra. For MS scans, the m/z scan range was 350–1,550. The fixed first mass was set as 100 m/z.

The resulting MS/MS data were processed using MaxQuant with the integrated Andromeda search engine (v.1.5.2.8). Tandem mass spectra were searched against *A. japonicus* transcriptome and genome databases (Sun et al., 2011; Du et al., 2012; Zhang et al., 2017) concatenated with the reverse decoy database. Trypsin/P was specified as the cleavage enzyme, allowing up to two missed cleavages, five modifications per peptide, and five charges. The mass error was set to 10 ppm for precursor ions and 0.02 Da for fragment ions. Carbamidomethylation on Cys was specified as a fixed modification, and oxidation on Met, phosphorylation on Ser, Thr, Tyr, and acetylation on the protein N-terminus were specified as variable modifications. The false discovery rate (FDR) threshold for proteins, peptides, and modification sites was 1%. Minimum peptide length was set at seven residues. For the quantification method, TMT-6plex was selected. All other parameters in MaxQuant were set to default values. The site localization probability was set as >0.5. We checked the mass error of all identified peptides, and the distribution was close to zero, and most were less than 0.02 Da, confirming that the mass accuracy of the MS data was acceptable. We next verified the length of most peptides was between eight and 20 residues, consistent with the properties of tryptic peptides, confirming that sample preparation conformed to the required standard.

A *p* < 0.05 from *t*-tests and a fold-change >1.50 or < 0.67 were set as the thresholds for differential phosphorylation. Because of the relatively lower protein recognition ration in acetylation study, a *p* < 0.05 by the *t*-test and a fold-change >1.20 or < 0.83 were set as the thresholds for differential acetylation to get a comprehensive understanding.

### Bioinformatics analysis

Gene ontology (GO; http://www.geneontology.org/) and Kyoto Encyclopedia of Genes and Genomes (KEGG; http://www.genome.jp/kegg/) databases were used to classify and group the identified proteins, and hypergeometric tests were used to define significantly enriched GO terms and pathways of differentially expressed proteins. A *p* < 0.05 was considered significant.

Domain annotation was performed using InterProScan (a sequence analysis application) based on the protein sequence alignment method. InterPro (http://www.ebi.ac.uk/interpro/) is a database that integrates diverse information about protein families, domains, and functional sites, and makes it freely available to the public via Web-based interfaces and services. Central to the database are diagnostic models, known as signatures, against which protein sequences can be searched to determine their potential functions. InterPro has utility in the large-scale analysis of whole genomes and meta-genomes, as well as in characterizing individual protein sequences.

### Pan acetylation western blotting

The same extracted proteins discussed above in section Protein Extraction and Trypsin Digestion were used in this experiment. Western blotting samples containing equal amounts of protein (20 μg/condition) were prepared by heating in 12% polyacrylamide buffer for 10 min at 95°C. Gel electrophoresis was then performed initially for 30 min at 80 V, then at 120 V until the bromophenol blue had run off the front of the gel. Following transfer to PVDF membranes (Millipore, Bedford, MA, USA) for 2 h at 80 V and 4°C, the membranes were blocked in phosphate-buffered saline (PBS) containing 5% non-fat milk and 0.1% tween-20 for 1 h at RT. Antibody incubation was performed by rinsing with TBST for 10 min three times, incubating at 4°C overnight with primary antibody (anti-acetyllysine antibody, PTM-101) diluted 1:1,000 in PBS with 1% non-fat powdered milk and 0.1% tween-20, rinsing with TBST for 10 min three times, incubating for 1 h at RT with secondary antibody (Thermo, Pierce, goat anti-mouse IgG, H+L, peroxidase-conjugated, 31430) diluted 1:5,000 in PBS with 1% non-fat powdered milk and 0.1% Tween-20), rinsing with TBST for 10 min three times, and staining with ECL western blot detection reagent (Beyotime, Beijing, China) followed by quantification using NIH Image 1.63 software (Syngene, Cambridge, UK).

### Results

#### Overview of phospho- and acetyl proteomics data

All acquired data are available via ProteomeXchange under identifier PXD008374. In this study, we quantified dynamic changes in phosphorylation during intestine regeneration in *A. japonicus*, identifying 2,584 phosphorylation sites in 1,531 proteins, among which 1,862 phosphorylation sites in 1,169 proteins were quantified (Table 1). In all, 127 phosphorylation sites were upregulated in 113 proteins, and 38 phosphorylation sites were downregulated in 34 proteins (Table 1, Data Sheet 1).

**Table 1:** Summary of identified, quantified, and differentially quantified sites and proteins of phosphorylation and acetylation.

	**Name**	**Identified**	**Quantified**	**Up-regulated**	**Down-regulated**
**Phosphorylation**	Modified sites	2,584	1,862	127	38
	Modified proteins	1,531	1,169	113	34
**Acetylation**	Modified sites	886	712	130	211
	Modified proteins	555	470	101	150

We also quantified the dynamic changes in acetylation during intestine regeneration in *A. japonicus*, and identified 886 acetylation sites in 555 proteins, among which 712 acetylation sites in 470 proteins were quantified (Table 1). In all, 130 acetylation sites were upregulated in 101 proteins, and 211 acetylation sites were downregulated in 150 proteins (Table 2, Data Sheet 2).

**Table 2:** Differentially phosphorylated proteins during intestine regeneration in sea cucumbers.

**Protein accession**	**Amino acid**	**Protein description (Nr database)**	**Modified sequence**	**Modified ratio**	***P*-value**	**Protein expression**	

						**Mean**	**SD**
**CYTOSKELETAL PROTEINS**							
Unigene13550_All	S	Tubulin alpha-1B chain-like	S(0.999)IQFVDWCPT(0.001)GFK	0.517	2.43E-02	0.756	0.007
CL128.Contig5_All	S	Tubulin beta chain-like	AVLVDLEPGTMDS(1)VR	2.224	8.71E-03	1.280	0.140
	S		IMNTY(0.004)S(0.002)VVPS(0.994)PK	2.199	4.27E-02		
	S		MS(1)MKEVDEQMLNVQNK	1.512	3.23E-03		
CL2542.Contig6_All	S	Neural alfa2 tubulin	SFNTFFS(0.001)ET(0.092)GS(0.907)GK	2.228	4.14E-03	1.456	0.263
CL2946.Contig3_All	S	Unconventional myosin-XVI, partial	KGS(1)IAPSIR	1.675	4.98E-03	1.024	0.424
CL9013.Contig2_All	T	Plastin 3-like	AFVT(1)PNDIVK	1.505	1.49E-02	1.558	0.193
Unigene27095_All	S	Myosin-IIIb-like	TGT(0.001)LALQNRIS(0.999)QK	1.657	1.15E-02	1.187	0.462
Unigene638_All	S	Microtubule-associated protein RP/EB family member 1	TQIS(1)ELSGELTTAR	1.612	4.29E-02	0.549	0.256
CL1337.Contig7_All	S	Lethal giant larvae homolog 1-like	S(0.965)S(0.035)SENMISLVR	1.564	4.35E-02	0.933	0.280
**PROTEIN SYNTHESIS AND MODIFICATION**							
**Transcription, RNA processing, and modification**							
CL9265.Contig2_All	S	CASP-like transcription factor	LDPFNS(1)FSR	2.454	5.63E-03	0.919	0.180
CL859.Contig2_All	S	Pre-mRNA-processing-splicing factor 8	AIS(0.995)AT(0.005)NLHLR	1.724	1.59E-02	1.243	0.170
CL5050.Contig1_All	S	Neuroblast differentiation-associated protein AHNAK-like	GPS(1)LKGGADVEIPSGK	2.257	3.06E-03	0.634	0.036
**Translation, ribosomal structure, and biogenesis**							
CL1510.Contig1_All	Y	Golgi integral membrane protein 4	AENELGQQY(1)FLQLR	3.55	6.98E-04	1.971	0.264
CL11778.Contig2_All	T	Golgi reassembly-stacking protein 2-like	S(0.021)FPET(0.953)PVDLS(0.026)QHMK	1.683	1.51E-02	1.706	0.117
CL11977.Contig3_All	S	Eukaryotic translation initiation factor 4E binding protein	HEY(0.027)S(0.066)T(0.338)T(0.338)PGGT (0.372)MFS(0.546)T(0.156)T(0.156)PGGT (0.001)R	0.659	9.92E-03	0.877	0.270
Unigene26560_All	Y	Ribosomal protein S10-like	GPSGPVDSQRDS(0.118)Y(0.882)R	0.637	3.21E-02	0.520	0.203
CL4911.Contig2_All	S	Methyltransferase fibrillarin-like	VYVEPHRLS(1)GVFIAR	1.794	2.80E-02	0.750	0.293
CL609.Contig1_All	S	Heterogeneous nuclear ribonucleoprotein U-like protein 1-like	NY(0.019)S(0.002)GRFDS(0.979)LIEK	1.892	8.20E-03	0.857	0.244
CL7370.Contig4_All	S	Ribosome-binding protein 1-like isoform 2	EKPGKS(1)PR	1.91	4.75E-03	0.385	0.131
CL10446.Contig2_All	S	Nucleic acid-associated protein 36	KRPS(0.886)S(0.057)T(0.057)NDGPAK	1.933	1.70E-02	0.428	0.216
Unigene8144_All	S	polyA-binding protein	LFS(1)IIQQSHGDVAGK	1.653	4.22E-03	0.719	0.229
CL6103.Contig1_All	S	Eukaryotic peptide chain release factor GTP-binding subunit ERF3B isoform 2	T(0.009)VEVGRAS(0.986)FET(0.004)DKK	1.575	4.16E-03	1.097	0.045
CL9576.Contig3_All	S	mRNA turnover protein 4 homolog	DKKVS(0.933)LT(0.063)ET(0.004)K	1.547	3.37E-02	0.637	0.286
CL9627.Contig2_All	S	Eukaryotic translation initiation factor 4B-like	RKDNVVS(1)PR	1.757	2.55E-02	0.962	0.127
Unigene1047_All	S	Putative 60S ribosomal protein RPL15	QAMS(1)LRR	1.712	4.10E-02	1.296	0.428
Unigene33503_All	T	60S ribosomal protein L27-like isoform 1	DVFREPT(1)LKK	2.68	1.35E-02	1.124	0.252
Unigene3874_All	S	40S ribosomal protein S13	S(1)VPNWLK	1.695	3.23E-03	0.659	0.171
Unigene46531_All	S	40S ribosomal protein S27-like isoform 3	LTEGCS(1)FR	1.788	2.66E-03	0.661	0.103
Unigene5010_All	S	Nucleosome assembly protein homolog	LDS(1)LVGQR	1.726	8.53E-03	0.823	0.221
Unigene53143_All	T	Translation initiation factor 2 subunit alpha (AeIF-2a)	KVHQT(1)VR	1.513	2.63E-02	0.870	0.123
Unigene7928_All	S	Heterogeneous nuclear ribonucleoprotein A1-like	FGS(0.999)VS(0.001)AVK	1.761	2.30E-05	0.908	0.250
Unigene26544_All	S	Histone H1.0-like	KLS(0.991)ES(0.009)QVK	1.597	9.24E-04	0.901	0.259
Unigene29854_All	Y	High mobility group protein 1 homolog	NLSAFFLY(1)SNDER	1.717	1.34E-02	0.638	0.194
Unigene32660_All	S	Heterogeneous nuclear ribonucleoprotein K-like	DLAGS(1)IIGTR	1.778	4.63E-02	0.524	0.173
**Posttranslational modification, protein turnover, chaperones**							
CL5303.Contig3_All	S	Peroxiredoxin 6-like	AGGDCMVLPS(1)VKAEDIPALFPK	2.157	3.09E-02	0.751	0.263
Unigene22155_All	S	E3 ubiquitin-protein ligase UBR4, partial	TQATIS(0.002)LAS(0.975)PT(0.019)ERPQS (0.002)S(0.002)QK	1.563	1.80E-02	1.059	0.495
Unigene25579_All	S	Heat shock protein 75 kDa, mitochondrial-like	LHS(1)FMEK	1.639	1.05E-02	0.994	0.148
Unigene15415_All	S	Polypeptide N-acetylgalactosaminyltransferase 10-like	AT(0.002)DPVRS(0.998)PIMAGGLFAIDR	1.871	2.79E-02	1.205	0.058
**SIGNAL RECOGNITION AND TRANSDUCTION**							
CL11595.Contig1_All	S	Tyrosine kinase receptor Cad96Ca-like	IEAISIQS(0.958)S(0.042)K	0.581	4.36E-03	0.641	0.185
Unigene32568_All	S	TBC1 domain family member 1-like	KHY(0.006)S(0.994)CEDQLDR	0.591	2.15E-02	1.870	0.883
Unigene9348_All	S	Sterile alpha and TIR motif-containing protein 1-like	VFDS(0.948)PT(0.052)RPK	0.615	4.59E-02	1.686	0.929
CL6832.Contig3_All	S	Epidermal growth factor receptor substrate 15-like 1-like	DDPFAS(0.911)FS(0.075)S(0.01)S(0.003) S(0.001)K	1.615	1.37E-02	1.127	0.342
CL7251.Contig2_All	S	TBC1 domain family member 24-like	S(0.26)S(0.737)PQS(0.002)LS(0.001)VPIA	1.668	1.48E-02	0.932	0.113
Unigene12365_All	S	Putative calcium-binding protein p22	FIS(0.994)LDKS(0.006)EAGK	2.775	1.95E-02	0.833	0.144
CL2850.Contig5_All	S	Ras-related GTP binding protein D	MSPNETLFLES(0.955)T(0.045)NK	1.968	1.89E-02	1.075	0.436
CL3382.Contig2_All	Y	tyrosine-protein kinase HTK16-like	ATGANNDY(0.004)Y(0.996)R	0.502	6.36E-03	0.802	0.038
CL6145.Contig1_All	Y	Low quality protein: tyrosine-protein kinase Tec-like	YVLDDQY(1)TSSGAR	0.561	3.19E-02	1.021	0.438
CL12103.Contig1_All	S	Rho guanine nucleotide exchange factor 7-like isoform 1	ELS(0.001)PNT(0.069)LS(0.931)PR	1.514	1.20E-02	1.041	0.234
CL5733.Contig2_All	T	Calcium/calmodulin-dependent protein kinase (CaM kinase) II delta-like isoform 1	QET(1)VDCLKK	1.802	3.72E-02	0.557	0.101
Unigene15296_All	S	FERM domain-containing protein 5-like	RS(1)GS(1)FILK	1.511	1.53E-03	0.768	0.051
Unigene3880_All	T	Oryzias latipes 14-3-3 protein beta/alpha-1-like	ADT(1)PKVELNPDELAK	1.517	3.04E-02	0.688	0.262
Unigene21911_All	T	Spectrin alpha chain, brain-like isoform 2	QET(1)FDAGLQSFEK	2.176	4.36E-02	1.059	0.123
CL8872.Contig1_All	Y	Proto-oncogene tyrosine-protein kinase Src	LVKDDHY(1)LAR	0.467	1.58E-02	0.825	0.007
CL9595.Contig1_All	S	Titin-like	SYPDS(0.009)S(0.008)NVRQS(0.965)PS (0.013)S(0.003)HTR	0.553	5.51E-04	1.097	0.176
	S		RGPADS(1)PIGR	0.561	2.33E-04		
	Y		RTES(0.002)PS(0.003)Y(0.995)R	0.627	8.89E-03		
	S		Y(0.007)HDDDRS(0.986)Y(0.007)DR	0.599	3.79E-03		
CL5942.Contig1_All	S	PKG	S(0.002)S(0.042)S(0.955)DS(0.001)VEST	2.483	1.29E-03	1.416	0.418
	T		T(0.068)WT(0.924)FCGT(0.008)PEYVAP	3.490	5.03E-03		
	T		T(0.037)WT(0.349)FCGT(0.614)PEYVAP	4.720	7.11E-03		
Unigene270_All	S	Protein kinase, cAMP-dependent, catalytic, beta a-like	IKGPGDTSHFDDYEEEPIRIS(0.905)S(0.073) T(0.022)EK	2.227	1.13E-02	0.837	0.026
**ENERGY PRODUCTION AND CONVERSION**							
CL2205.Contig3_All	S	ATP-citrate synthase	KPAS(0.998)FMT(0.002)SIVDER	1.558	3.89E-02	1.260	0.272
Unigene200_All	S	Aldehyde dehydrogenase 3 family, member A2	PVPS(1)ELVMGIR	3.812	1.89E-02	1.289	0.257
CL2397.Contig1_All	S	ATP-binding cassette sub-family D member 3-like	RKS(1)T(1)FADLK	0.463	4.36E-02	0.708	0.103
**SUBSTANCE TRANSPORT AND METABOLISM**							
**Amino acid transport and metabolism**							
CL5840.Contig2_All	S	Glutamine synthetase	GAS(1)IRIPR	3.302	1.54E-03	1.128	0.212
CL11928.Contig1_All	S	Peroxisomal sarcosine oxidase-like	KNS(1)EPAIIEK	0.201	2.92E-03	0.311	0.089
CL6111.Contig2_All	S	Protein transport protein Sec31A-like isoform 1	RPAGAS(1)FGFGGK	1.749	4.75E-03	1.710	0.400
	S		S(0.209)KS(0.79)LEEALS(0.001)QGQFC	4.286	3.36E-02		
Unigene32921_All	S	Probable thiopurine S-methyltransferase-like	MFAWCS(0.82)S(0.18)IK	2.859	2.12E-03	3.157	0.481
**Coenzyme transport and metabolism**							
CL12230.Contig2_All	S	Small ubiquitin-related modifier 3	VVGS(0.003)EGS(0.947)T(0.05)VQFK	1.701	5.75E-03		
Unigene29548_All	S	NADH-cytochrome b5 reductase-like	S(0.01)MDY(0.021)T(0.01)S(0.011)CVLDS (0.013)IQAVS(0.645)T(0.645)DT(0.645)F	0.645	2.83E-02	1.047	0.036
	T		S(0.01)MDY(0.021)T(0.01)S(0.011)CVLDS (0.013)IQAVS(0.645)T(0.645)DT(0.645)F	0.645	2.83E-02		
	T		S(0.01)MDY(0.021)T(0.01)S(0.011)CVLDS (0.013)IQAVS(0.645)T(0.645)DT(0.645)F	0.645	2.83E-02		
**Lipid transport and metabolism**							
CL8209.Contig2_All	S	Acetyl-Coenzyme A acyltransferase 2-like	TPFGAFGGS(1)LK	1.774	2.20E-02	0.957	0.330
CL3144.Contig1_All	S	Oxysterol-binding protein-related protein 11-like	YS(0.012)S(0.976)S(0.011)ESIGGR	0.635	4.01E-02	0.802	0.072
CL2380.Contig1_All	S	Niemann-Pick type C1 domain-containing protein	HPGS(1)KAQLEDVWEK	3.795	6.02E-04	2.317	0.276
**Inorganic ion transport and metabolism**							
CL4987.Contig2_All	S	Catalase	LT(0.012)T(0.042)S(0.502)S(0.445)GCPID	0.624	9.00E-03	0.881	0.100
CL574.Contig3_All	T	Sarco/endoplasmic reticulum calcium transporting ATPase	VGEAT(0.965)ET(0.035)ALTVLVEK	2.423	1.59E-02	0.772	0.200

#### GO, domain, KEGG pathway, and subcellular localization analyses

**Figure 2:**
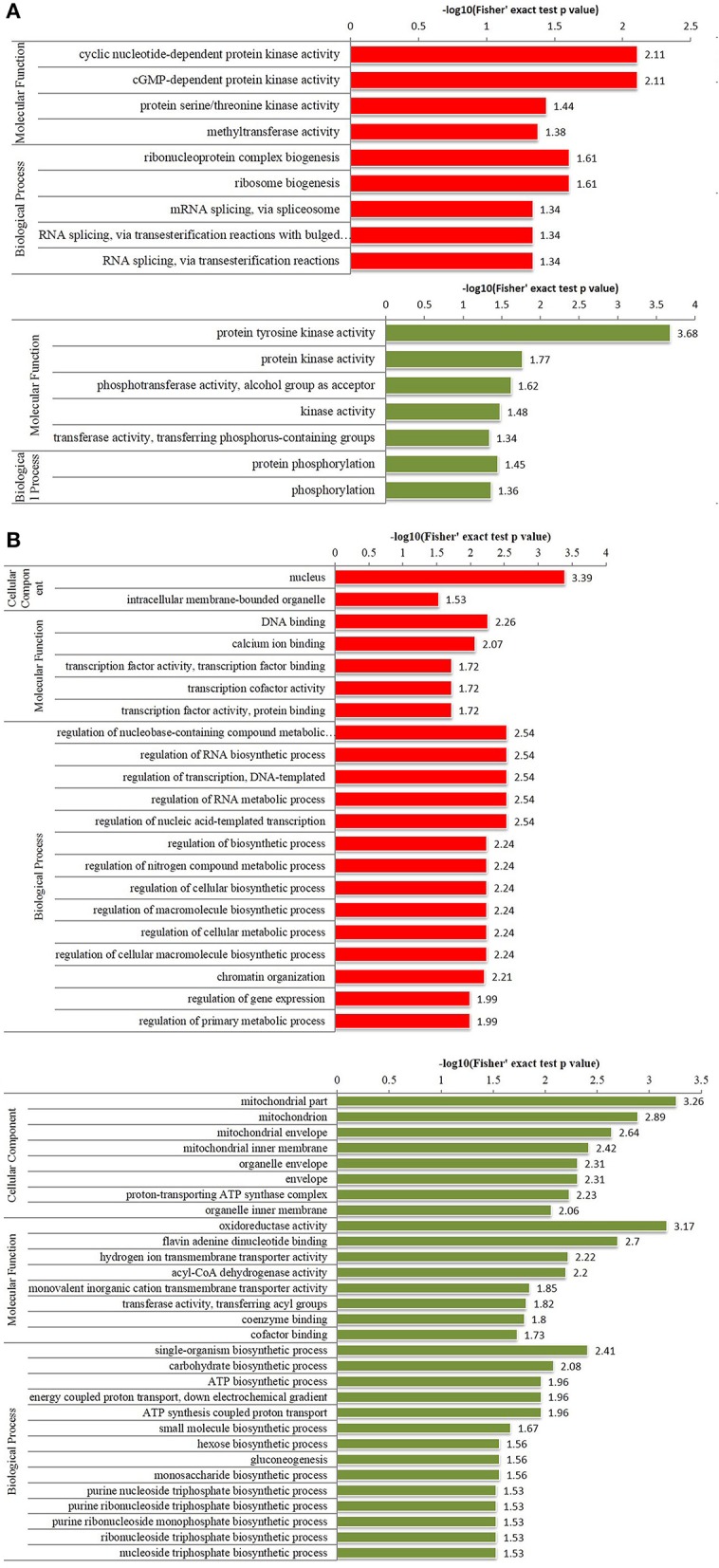
Enriched gene ontology (GO) analysis of differentially phosphorylated and acetylated proteins (*p* < 0.05). **(A)** phosphorylation. **(B)** Acetylation. Red, GO terms of proteins for which the modification was upregulated. Green, GO terms of proteins for which the modification was downregulated.

**Figure 3:**
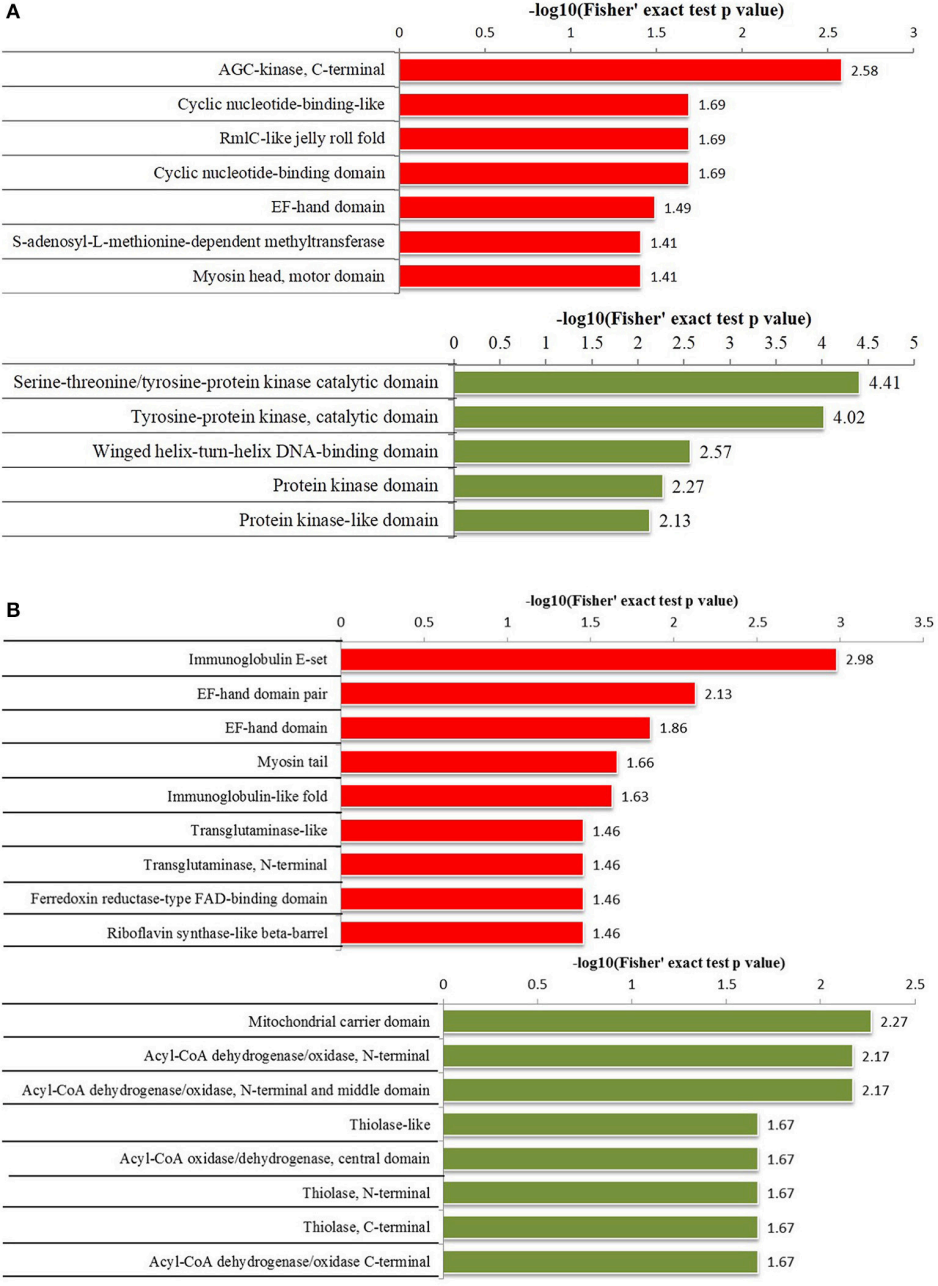
Protein domain enrichment analysis of differentially phosphorylated and acetylated proteins (*p* < 0.05). **(A)** phosphorylation. **(B)** Acetylation. Red, Protein domain terms for which the modification was upregulated. Green, Protein domain terms for which the modification was downregulated.

**Figure 4:**
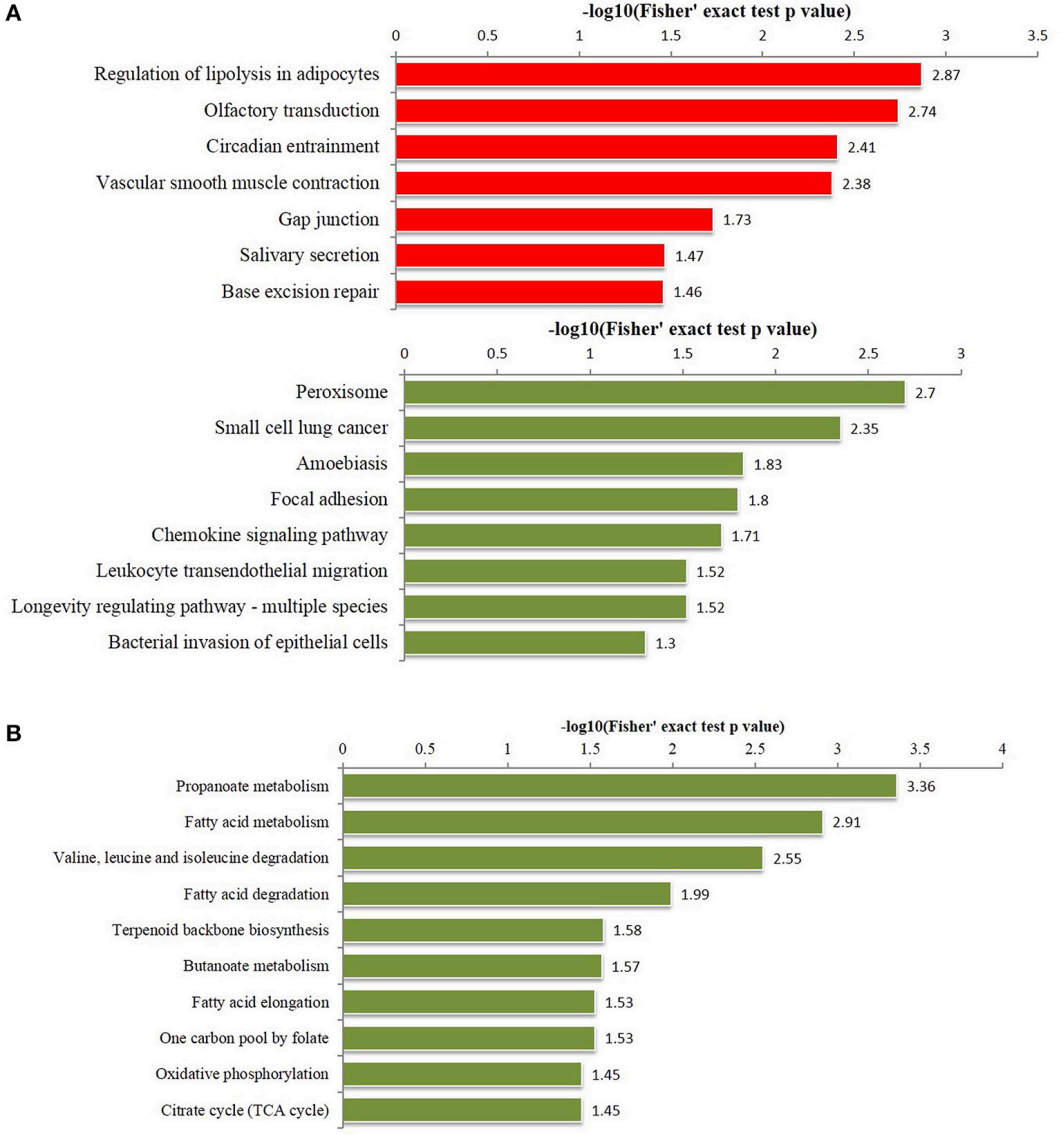
Enriched pathways analysis of differentially phosphorylated and acetylated proteins (all *p* < 0.05). **(A)** phosphorylation. **(B)** Acetylation. Red, Pathways terms of proteins for which the modification was upregulated. Green, Pathways terms of proteins for which the modification was downregulated.

**Figure 5:**
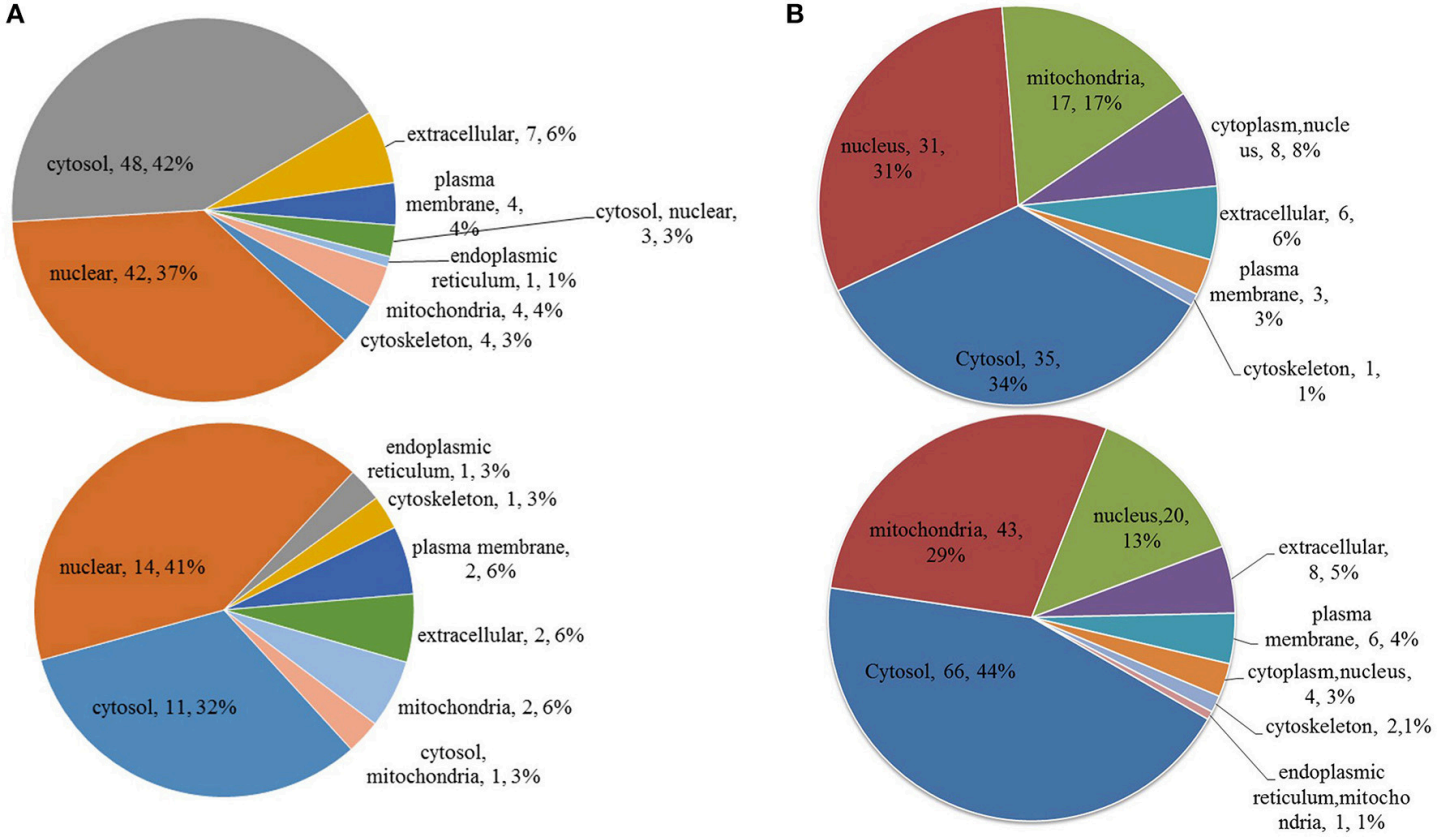
Subcellular location of differentially phosphorylated and acetylated proteins. **(A)** phosphorylation. **(B)** acetylation. Up, subcellular location of proteins for which the modification was upregulated. Down, subcellular location of proteins for which the modification was downregulated.

To further understand the functions and features of the differentially phosphorylated and acetylated proteins, we annotated data based on GO (Figure 2), domains (Figure 3), pathways (Figure 4), and subcellular localization (Figure 5).

GO analysis showed significant enrichment of upregulated phosphorylated proteins in 4 terms of molecular function (MF) including cyclic nucleotide-dependent protein kinase activity and cGMP-dependent protein kinase activity etc., and five terms of biological process (BP), including ribonucleoprotein complex biogenesis, and ribosome biogenesis etc. (Figure 2A). Downregulated phosphorylated proteins were significantly enriched in 5 terms of MF including protein tyrosine kinase activity and protein kinase activity etc., and in two terms of BP including protein phosphorylation and phosphorylation (Figure 2A). Upregulated acetylated proteins were enriched in 2 terms of cellular component (CC) including nucleus and intracellular membrane-bounded organelle, five terms of MF including DNA binding and calcium ion binding etc., and 14 terms of BP including regulation of nucleobase-containing compound metabolic processes, and regulation of RNA biosynthetic processes etc. (Figure 2B). Downregulated acetylated proteins were enriched in 8 terms of CC including mitochondrion and organelle envelope etc., eight terms of MF including oxidoreductase activity and flavin adenine dinucleotide binding etc., and 14 terms of BP including single organism biosynthetic processes, and carbohydrate biosynthetic processes etc. (Figure 2B).

Protein domain enrichment analysis revealed that upregulated phosphorylated proteins were significantly enriched in 7 terms including AGC-kinase, C-terminal, cyclic nucleotide-binding-like, and RmlC-like jelly roll fold etc., while downregulated phosphorylated proteins were significantly enriched in 5 terms including the serine-threonine/tyrosine-protein kinase catalytic domain, tyrosine-protein kinase catalytic domain, and winged helix-turn-helix DNA-binding domain etc. (Figure 3A). Upregulated acetylated proteins were significantly enriched in 9 terms including immunoglobulin E-set, EF-hand domain pair, and myosin tail etc., while downregulated acetylated proteins were significantly enriched in 8 terms including mitochondrial carrier domain, and Acyl-CoA dehydrogenase/oxidase N-termini etc. (Figure 3B).

KEGG pathway-based enrichment analysis revealed 7 pathways for upregulated phosphorylated proteins including regulation of lipolysis in adipocytes, olfactory transduction, and circadian entrainment, while 8 pathways for downregulated phosphorylated proteins included peroxisome, small cell lung cancer, and amoebiasis (Figure 4A). Regarding acetylation, no pathways were significantly enriched for upregulated proteins, but 10 pathways were identified for downregulated acetylated proteins including propanoate metabolism, fatty acid metabolism, and valine, leucine and isoleucine degradation (Figure 4B).

Subcellular location annotation information of differentially phosphorylated and acetylated proteins is shown in Figure 5. Regarding phosphorylation, most up- and downregulated proteins were mainly localized to the nucleus and cytosol (Figure 5A). For acetylation, most upregulated proteins were localized to the nucleus and cytosol, whereas most downregulated proteins to the cytosol and mitochondria (Figure 5B).

#### Differentially phosphorylated and acetylated proteins

**Figure 6:**
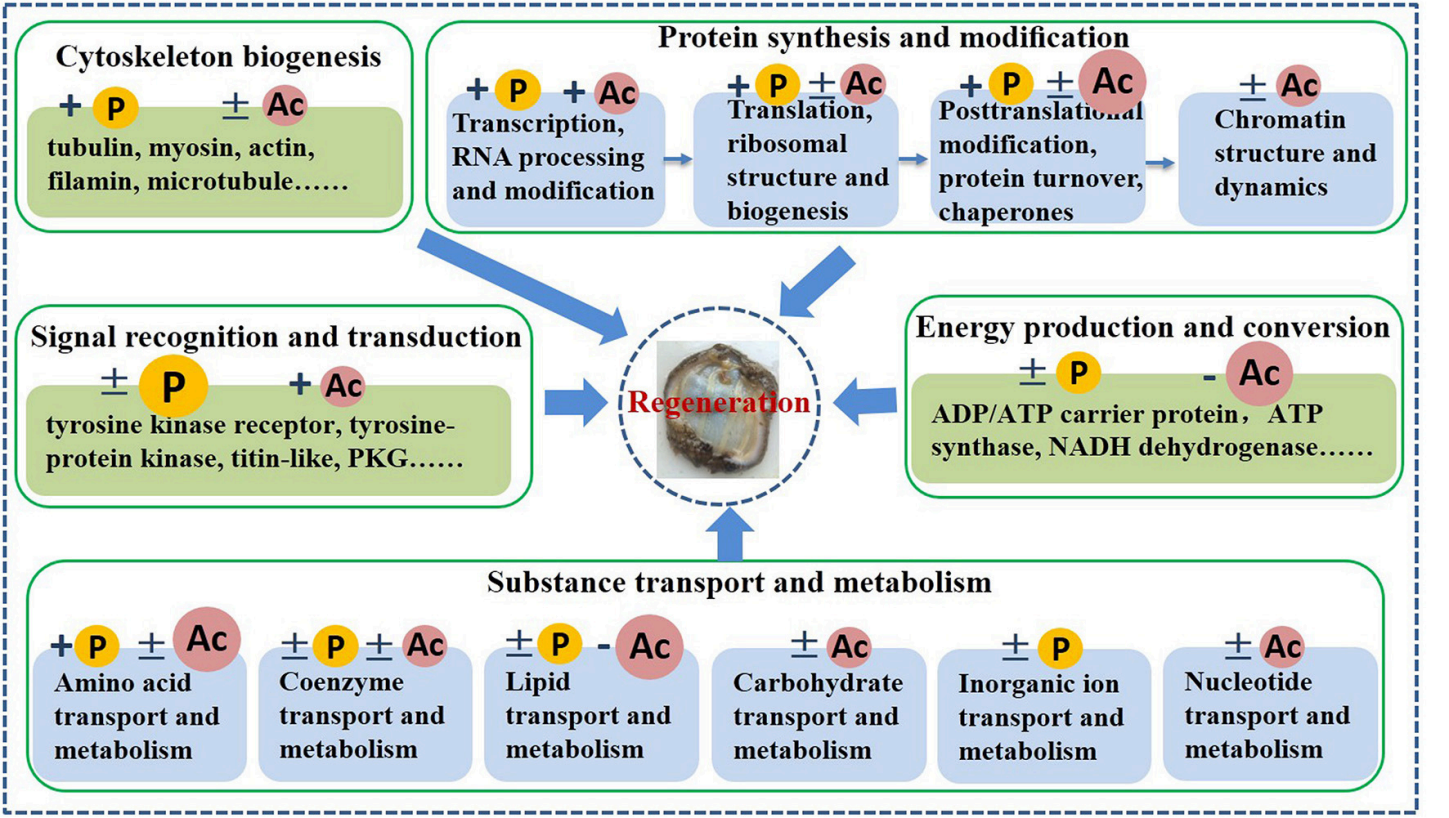
Schematic model of phosphorylation and acetylation during intestine regeneration in sea cucumbers. +, modification levels of almost all proteins were upregulated; −, modification levels of almost all proteins were downregulated; ±, modification levels of proteins were up- or downregulated. P, phosphorylation; Ac, acetylation; Big P or Ac, a large number of proteins were regulated by this reversible modification.

Proteins differentially phosphorylated and acetylated during intestine regeneration were mainly related to cytoskeleton biogenesis (P: 8; A: 14), protein synthesis and modification (P: 29; A: 44), signal recognition and transduction (P: 18; A: 4), energy production and conversion (P: 3; A: 30), and substance transport and metabolism (P: 11; A: 58) (Tables 2, 3, Figure 6). Phosphorylated sites were mainly serine (S), followed by tyrosine (Y) and threonine (T). Most acetylated sites were lysine (K). Further studies showed the proteins regulate “protein synthesis and modification” at different levels, including transcription factors, RNA processing and modification (P: 3, A: 2), translation, ribosomal structure and biogenesis (P: 22, A: 18), posttranslational modification, protein turnover, chaperones (P: 4, A: 15), and chromatin structure and dynamics (A: 9; Tables 2, 3, Figure 6). All differentially modified proteins “in substance transport and metabolism” were divided into six groups: amino acid transport and metabolism (P: five; A:25,), coenzyme transport and metabolism (P:2; A: 2), lipid transport and metabolism (P:3; A: 25), carbohydrate transport and metabolism (A:4), inorganic ion transport and metabolism (P:2), and nucleotide transport and metabolism (A: 2; Tables 2, 3, Figure 6). Moreover, most of the phosphorylated proteins played important roles in signal recognition and transduction. Whilemost acetylated proteins were related to posttranslational modification, protein turnover, chaperones (15), energy production and conversion (30), as well as amino acid and lipid transport and metabolism (25). Additionally, in order to investigate the significance of the observed phosphorylation and acetylation, the results were compared with our previous protein expression data (Sun et al., 2017c).

**Table 3:** Differentially acetylated proteins during intestine regeneration in sea cucumbers.

**Protein accession**	**Amino acid**	**Protein description (Nr database)**	**Modified sequence**	**Modified ratio**	***P*-value**	**Protein expression**	

						**Mean**	**SD**
**CYTOSKELETAL PROTEINS**							
mRNA.AJAP19862	K	Actin, cytoskeletal	EITALAPPTMK(1)IK	1.494	1.08E-02	0.917	0.166
	K		IK(1)IIAPPER	0.810	1.24E-02		
mRNA.AJAP28693	K	Actin, non-muscle 6.2	EISALAPPTMK(1)IK	1.384	8.67E-05	/	/
mRNA.AJAP11142	K	Myosin heavy chain, embryonic smooth muscle isoform (Fragment)	LESAK(1)LHLER	1.228	2.68E-03	0.906	0.317
mRNA.AJAP05801	K	Myosin heavy chain, striated muscle	EQLDK(1)FNTR	1.233	1.03E-35	0.894	0.052
	K		TSSEALK(1)TIK	1.234	1.45E-03		
	K		K(1)THDGLR	1.306	9.99E-03		
mRNA.AJAP25461	K	Myosin regulatory light chain 2, smooth muscle minor isoform	MIK(1)HGTQDESLVK	1.241	2.10E-13	1.480	0.276
mRNA.AJAP29917	K	Alpha-actinin-1	K(1)HIAFESDLAAHQDR	0.690	6.54E-12	0.970	0.077
	K		LDHLAQK(1)FK	0.627	6.86E-04		
mRNA.AJAP21855	K	F-actin-capping protein subunit alpha	VATDSQSGSVDK(1)TAEPWR	0.780	9.11E-18	/	/
mRNA.AJAP23465	K	Filamin-A	FVPTQDGVHTVSVK(1)NK	1.325	2.01E-49	/	/
	K		AEIK(1)FQDNK	1.387	9.38E-08		
	K		YGGQNHIGGSPFLAHIK(1)GTPK	1.211	1.29E-03		
	K		EK(1)GNYWLVVK	1.511	8.92E-03		
mRNA.AJAP01874	K	Filamin-C	ITPTFAGQPLGK(1)SQGAPVGK	1.636	2.07E-25	0.737	0.360
	K		YGGDNAGNSPYSVK(1)VVPTGDA	1.534	1.11E-03		
mRNA.AJAP24084	K	Fimbrin	AFVTPNDIVK(1)GNSK	1.386	4.93E-07	1.558	0.193
	K		IK(1)NFSGDIK	1.293	8.01E-03		
mRNA.AJAP28261	K	Microtubule-actin cross-linking factor 1	QTK(1)HEHCAK	0.611	1.36E-02		
mRNA.AJAP19671	K	Myosin regulatory light chain 12B	ILK(1)HGTK	1.213	7.51E-03	0.684	0.230
mRNA.AJAP23147	K	Myosin-11	LHKEEK(1)R	1.211	3.20E-03	0.797	0.103
mRNA.AJAP21857	K	Nuclear migration protein nudC	K(1)TDFYQGALK	1.244	2.04E-02	0.715	0.144
**PROTEIN SYNTHESIS AND MODIFICATION**							
**Transcription, RNA processing, and modification**							
mRNA.AJAP29853	K	Histone acetyltransferase p300	GGK(1)GGGK(1)GGGK(1)GPMEDK	1.366	1.48E-20	1.033	0.048
	K		IVDVK(1)SGMR	1.390	9.24E-05		
	K		K(1)SNK(1)SK(1)SGTQR	1.675	1.62E-04		
	K		LYATMEK(1)HK	1.412	1.52E-07		
	K		QPGGSAPPSADPGPQPGDK(1)LAA	2.143	4.89E-04		
mRNA.AJAP27535	K	Poly [ADP-ribose] polymerase 1	NHFK(1)DLYLEK	1.314	2.45E-03	1.271	0.278
**Translation, ribosomal structure, and biogenesis**							
mRNA.AJAP05187	K	28S ribosomal protein S22, mitochondrial“	VYEHIK(1)K	0.762	2.53E-03	/	/
mRNA.AJAP25592	K	39S ribosomal protein L12, mitochondrial”	ADIGK(1)DEASK	0.732	7.58E-03	/	/
mRNA.AJAP01527	K	40S ribosomal protein S19	AASEGKPAAK(1)H	1.637	3.18E-03	0.838	0.159
mRNA.AJAP25251	K	40S ribosomal protein S3a	K(1)TSYAQTQQVR	1.357	1.89E-03	/	/
mRNA.AJAP18554	K	60S ribosomal protein L21	VK(1)NNTHGVR	1.259	1.10E-03	1.355	0.632
mRNA.AJAP13674	K	60S ribosomal protein L23a	EPASK(1)VEAR	1.294	4.62E-03	/	/
mRNA.AJAP24211	K	60S ribosomal protein L28	MYNK(1)VTFK	1.264	9.71E-03	1.355	0.632
mRNA.AJAP06626	K	60S ribosomal protein L34-B	AFLIEEQK(1)IVHR	0.221	1.65E-05	1.015	0.267
mRNA.AJAP29607	K	60S ribosomal protein L35	VTGGQASK(1)LSK	1.326	1.66E-04	/	/
mRNA.AJAP18192	K	60S ribosomal protein L5	QGK(1)TDYHAR	1.255	6.94E-05	1.729	0.347
mRNA.AJAP27436	K	60S ribosomal protein L7	YALTYK(1)K	1.449	8.62E-03	/	/
mRNA.AJAP23305	K	Elongation factor Ts, mitochondrial“	SSPSGK(1)FTLGK	0.529	5.91E-04	/	/
mRNA.AJAP05683	K	Eukaryotic translation elongation factor 1 epsilon-1	YFNLK(1)NCK	0.801	1.46E-02	0.895	0.282
mRNA.AJAP23222	K	Eukaryotic translation initiation factor 5A	IVEMSTSK(1)TGK	0.751	7.33E-10	0.661	0.016
mRNA.AJAP00699	K	Glycine–tRNA ligase	AHLEK(1)LMK	0.718	1.63E-02	/	/
mRNA.AJAP18377	K	Leucine–tRNA ligase, mitochondrial”	LK(1)TSIPGVDPVLR	0.816	8.13E-03	/	/
mRNA.AJAP17380	K	Threonine–tRNA ligase, cytoplasmic“	EWK(1)HLQEEAAK	0.746	4.53E-05	1.062	0.151
mRNA.AJAP09019	K	Tryptophan–tRNA ligase, cytoplasmic”	LIEK(1)FGSK	1.258	8.29E-03	5.317	2.293
**Posttranslational modification, protein turnover, chaperones**							
mRNA.AJAP20021	K	[F-actin]-methionine sulfoxide oxidase MICAL2	K(1)FSESNDALEIK	15.173	1.22E-03	0.746	0.185
mRNA.AJAP19776	K	10 kDa heat shock protein, mitochondrial"	FK(1)PLFDR	0.693	3.13E-03		
mRNA.AJAP20333	K	26S protease regulatory subunit 6A-A	ACAAQTK(1)STFLK	1.419	3.27E-05	0.686	0.094
mRNA.AJAP26698	K	26S proteasome non-ATPase regulatory subunit 1	NLYSAAISDK(1)HEDIMAK	1.734	1.64E-04	1.133	0.439
mRNA.AJAP04803	K	E3 ubiquitin-protein ligase CBL-B	QTVDLFK(1)HSK	1.331	2.57E-03	/	/
mRNA.AJAP19853	K	Glutathione S-transferase theta-3	ITHIVQK(1)TFLGDNK	1.472	5.81E-03	0.917	0.238
mRNA.AJAP17671	K	Heat shock protein HSP 90-alpha 1	K(1)HLEVNPDHPIIETLR	1.568	1.53E-07	0.793	0.179
	K		VIK(1)DILDK	0.825	1.98E-02		
mRNA.AJAP27770	K	Maleylacetoacetate isomerase	ILK(1)FIGAER	2.404	2.73E-05	1.261	0.134
mRNA.AJAP17312	K	Peptidyl-prolyl cis-trans isomerase	TSK(1)K(1)IEIANCGK	0.493	3.83E-05	1.172	0.363
	K		IEGYGSQSGK(1)TSK	1.273	1.05E-12		
	K		KIEIANCGK(1)L	0.775	6.25E-03		
	K		IVMK(1)LEDAVVPK	0.644	1.51E-02		
	K		FADENFQLK(1)HK	0.780	2.11E-08		
	K		TSK(1)K(1)IEIANCGK	0.643	3.83E-05		
mRNA.AJAP09273	K	Peroxiredoxin-6	CVFIVGPDK(1)K	0.796	1.80E-02	0.751	0.263
mRNA.AJAP07241	K	Prefoldin subunit 1	TVLEDK(1)VK	1.260	1.74E-02	1.140	0.175
mRNA.AJAP18600	K	Proteasome subunit alpha type-6	QTEANSFLEK(1)K	1.281	1.19E-03	/	/
mRNA.AJAP14569	K	Protein disulfide-isomerase	AEDEK(1)FNVIHGEFQK	0.719	4.37E-03	2.305	0.867
mRNA.AJAP11875	K	S-crystallin SL11	ELLETEATK(1)HFK	0.415	1.10E-39	1.579	0.589
mRNA.AJAP16242	K	Thioredoxin	K(1)LAEEHTDVVFLK	1.279	1.18E-06	3.196	1.333
**Chromatin structure and dynamics**							
mRNA.AJAP27371	K	Histone H1, gonadal	GIVK(1)SNVIVNK	1.549	2.95E-05	/	/
mRNA.AJAP24569	K	Histone H2A.V	AGK(1)DSGK(1)AK(1)AK	1.279	2.35E-17	0.343	0.001
	K		AGK(1)DSGK(1)AK(1)AK	1.494	2.35E-17		
mRNA.AJAP27649	K	Histone H2AX	GK(1)GSK(1)SGSVAK	0.343	6.68E-04	0.999	0.895
mRNA.AJAP00708	K	Histone H2B	APK(1)AAGK(1)GAK(1)K	0.663	5.21E-03	1.348	0.214
mRNA.AJAP28539	K	Histone H3, embryonic	K(1)SAPATGGVK	0.772	3.43E-03	/	/
mRNA.AJAP14187	K	Histone H3.3	STGGK(1)APRK(1)QLATK(1)AAR	1.575	7.03E-35	0.882	0.246
	K		STGGK(1)APRK(1)QLATK(1)AAR	1.664	7.03E-35		
	K		K(1)STGGK(1)APR	1.201	1.43E-04		
	K		EIAQDFK(1)TELR	1.450	2.35E-05		
	K		YQK(1)STELLIR	1.290	2.19E-18		
mRNA.AJAP27350	K	Histone H4	DAVTYCEHAK(1)R	1.306	2.72E-13	/	/
mRNA.AJAP05921	K	Histone H5	TAAK(1)PPQHPK	1.304	4.68E-03	/	/
mRNA.AJAP02884	K	Structural maintenance of chromosomes protein 3	IETK(1)LEK	0.833	1.52E-02	/	/
	K		VIGAK(1)K(1)DQYILDK	1.505	2.61E-09		
**SIGNAL RECOGNITION AND TRANSDUCTION**							
mRNA.AJAP03399	K	Dual oxidase 1	TLEK(1)FFR	1.483	1.59E-02	/	/
	K		DEFK(1)HMLK	1.390	1.79E-02		
mRNA.AJAP26716	K	Twitchin	LVAK(1)SEYIFR	1.462	4.43E-03	0.620	0.236
mRNA.AJAP21880	K	Serine/threonine-protein phosphatase 4 regulatory subunit 2	ELPQTEPSEEPAAK(1)R	1.585	4.04E-03	0.845	0.019
mRNA.AJAP23904	K	Serine/threonine-protein phosphatase PP1-beta catalytic subunit	IYGFYDECK(1)R	1.202	9.73E-03	/	/
**ENERGY PRODUCTION AND CONVERSION**							
mRNA.AJAP17383	K	ADP,ATP carrier protein	YK(1)QLFLSGVDK	0.601	1.73E-02	0.809	0.206
mRNA.AJAP00498	K	ADP/ATP translocase 2	IAK(1)TEGGSAFFK	0.681	6.40E-21	/	/
mRNA.AJAP18006	K	ATP synthase F(0) complex subunit B1, mitochondrial	MPEHGGK(1)VR	0.812	1.99E-03	0.890	0.075
	K		CLTDLK(1)GLAK	0.747	1.38E-03		
mRNA.AJAP17777	K	ATP synthase subunit alpha, mitochondrial	GAFK(1)TTTR	0.527	7.86E-05	/	/
	K		VEGQINPETDAK(1)LK	0.755	5.35E-10		
	K		EGDVVK(1)R	0.688	3.54E-04		
	K		K(1)FLQHVK	0.523	1.19E-03		
	K		DNGK(1)HALIIYDDLSK	0.784	1.10E-04		
	K		GHLDK(1)LDPTK	0.763	4.49E-12		
mRNA.AJAP18495	K	ATP synthase subunit d, mitochondrial	K(1)TVPVAGLVDK	0.782	2.14E-03	0.798	0.132
	K		VSK(1)FVVESNNR	0.796	4.74E-09		
	K		TK(1)ADALK	0.753	1.52E-02		
mRNA.AJAP27569	K	ATP synthase subunit f, mitochondrial	DVK(1)LGQLPK	0.805	4.91E-03	0.781	0.082
mRNA.AJAP08412	K	ATP synthase subunit g, mitochondrial	TGK(1)FMNLTVK	0.717	5.56E-03	0.910	0.095
	K		GFSK(1)IVDSAK	0.771	9.70E-04		
mRNA.AJAP28616	K	ATP synthase subunit O, mitochondrial	K(1)LEQADSELK	0.495	1.92E-12	/	/
mRNA.AJAP10269	K	Chromate reductase	DSNDLPK(1)DLK	0.625	1.96E-03	/	/
	K		DLK(1)TAASQIK	0.805	7.00E-19		
mRNA.AJAP25225	K	Citrate synthase, mitochondrial	PK(1)SMSTEGLKK	0.814	5.67E-04	0.888	0.133
	K		VPETQADVK(1)EFR	0.714	4.09E-22		
mRNA.AJAP11350	K	Dihydrolipoyl dehydrogenase, mitochondrial	AK(1)TNADTDGLVK	0.710	8.50E-116	1.624	0.285
	K		SEEQLK(1)EEGVK	0.596	2.17E-04		
mRNA.AJAP05410	K	Electron transfer flavoprotein subunit alpha, mitochondrial	GLK(1)NGENFK	0.507	8.86E-03	1.208	0.131
mRNA.AJAP22586	K	Fumarate hydratase class I, aerobic	GGGSANK(1)TFLYQQTK	0.726	4.26E-14	0.895	0.144
	K		FVDEK(1)IK	0.752	1.84E-02		
	K		LMK(1)FVDEK	0.740	7.97E-03		
mRNA.AJAP13146	K	Inorganic pyrophosphatase	TDACGISLK(1)NVSVK	0.462	9.94E-25	1.447	0.311
mRNA.AJAP16353	K	Interferon-induced very large GTPase 1	ILEELCLK(1)K	70.988	1.87E-02	1.201	0.673
mRNA.AJAP29382	K	Isocitrate dehydrogenase [NADP], mitochondrial (Fragment)	YK(1)PDFEAK	0.389	7.56E-03	1.109	0.115
	K		LDNNEDLK(1)K	0.780	3.33E-07		
	K		AK(1)LDNNEDLKK	0.797	2.18E-04		
mRNA.AJAP18450	K	Isocitrate dehydrogenase [NADP], mitochondrial (Fragment)	FEIVFTPADGSK(1)K	0.669	4.92E-07	/	/
	K		VIWEK(1)IK	0.755	1.10E-02		
mRNA.AJAP19578	K	Mitochondrial 10-formyltetrahydrofolate dehydrogenase	VVEEIK(1)K	0.699	1.26E-02	0.958	0.258
	K		LVEEVK(1)QK	0.814	5.15E-03		
mRNA.AJAP00691	K	NAD(P) transhydrogenase, mitochondrial	ENTSMLLGDAK(1)K	0.580	8.58E-05	0.687	0.067
	K		K(1)TCDALLTQIR	0.733	6.77E-13		
mRNA.AJAP20861	K	NADH dehydrogenase [ubiquinone] 1 beta subcomplex subunit 11, mitochondrial	VQEIIDEGK(1)R	0.580	2.48E-04	0.963	0.267
mRNA.AJAP19755	K	NADH-cytochrome b5 reductase 3	VYFK(1)NVHPK	1.277	1.02E-03	1.047	0.036
mRNA.AJAP29907	K	Phosphoenolpyruvate carboxykinase [GTP], mitochondrial	K(1)TIISTQFK	0.300	7.17E-04	1.030	0.187
mRNA.AJAP26943	K	Probable pyruvate dehydrogenase E1 component subunit alpha, mitochondrial	RMETAAGTLYK(1)SK	1.390	5.55E-06	0.704	0.030
mRNA.AJAP07603	K	Pyruvate carboxylase, mitochondrial	FIGPSPK(1)VVHQMGDK	0.364	1.02E-03	1.998	0.548
	K		GK(1)ADEAYLIGK	0.618	7.79E-04		
mRNA.AJAP18370	K	Pyruvate dehydrogenase E1 component subunit beta, mitochondrial	K(1)TLHVDSALQTN	0.827	6.50E-04	0.704	0.030
mRNA.AJAP25640	K	Quinone oxidoreductase-like protein 2	SVEDVFK(1)LCDQGK	0.831	3.56E-04	0.410	0.109
	K		K(1)SVEDVFK	0.658	1.43E-02		
mRNA.AJAP20389	K	Succinate–CoA ligase [ADP-forming] subunit beta, mitochondrial (Fragment)	AEVAK(1)TSQQAYEIAK	0.800	1.06E-06	0.992	0.114
	K		ILACDDLDEAAK(1)K	0.688	1.42E-11		
mRNA.AJAP08012	K	Succinate–CoA ligase [GDP-forming] subunit beta, mitochondrial (Fragment)	QMELK(1)VPLVVR	0.525	1.42E-02	1.185	0.360
	K		LISSDTK(1)VK	0.727	2.42E-03		
mRNA.AJAP02331	K	Succinate-semialdehyde dehydrogenase, mitochondrial	DGQK(1)FLVK	0.624	2.11E-02	0.992	0.235
mRNA.AJAP08548	K	Sulfide: quinone oxidoreductase, mitochondrial	AFQNFK(1)K	0.724	1.16E-02	0.604	0.055
	K		SSPLAGPAGFMTVNK(1)HTGQHTK	0.732	2.37E-08		
**SUBSTANCE TRANSPORT AND METABOLISM**							
**Amino acid transport and metabolism**							
mRNA.AJAP22308	K	4-hydroxy-2-oxoglutarate aldolase, mitochondrial	HK(1)NIIALK	0.758	6.22E-03	0.702	0.094
mRNA.AJAP27694	K	Aminomethyltransferase, mitochondrial	K(1)TLLYDFHVEHGAK	0.647	1.94E-08	1.051	0.126
mRNA.AJAP19987	K	Aminomethyltransferase, mitochondrial	K(1)LADFPGASHVLTHLK	1.463	9.57E-03	/	/
mRNA.AJAP28106	K	Argininosuccinate synthase	IK(1)HDLSLR	0.763	5.81E-04	0.956	0.387
mRNA.AJAP01355	K	Asparagine synthetase	GLVDIQK(1)HK	0.655	2.11E-03	1.268	0.226
mRNA.AJAP09477	K	Aspartate aminotransferase, mitochondrial	EMSSIIK(1)NK	0.597	1.93E-03	0.865	0.129
	K		AEEILLSK(1)K	1.368	2.55E-02		
	K		VGSMFFK(1)K	0.512	1.08E-02		
	K		MK(1)NDHSVYLTR	0.830	3.05E-39		
	K		VGAFTFVCSSPDEMK(1)R	2.137	4.33E-03		
	K		HVGHEVK(1)SYR	0.533	2.49E-08		
mRNA.AJAP00354	K	Cysteine desulfurase, mitochondrial	ANK(1)VFFHTDAAQAVGK	1.388	5.68E-07	1.383	0.523
mRNA.AJAP20116	K	Delta-1-pyrroline-5-carboxylate synthase	SDLK(1)HCQK	0.408	3.60E-03	2.633	0.248
mRNA.AJAP00497	K	Glutamine–fructose-6-phosphate aminotransferase [isomerizing] 1	GDTDAVK(1)SASR	0.710	6.42E-06	/	/
mRNA.AJAP14703	K	Homocysteine S-methyltransferase 1	MLMTK(1)PELIK	1.386	5.67E-03	0.304	0.127
mRNA.AJAP05619	K	Lengsin	LAK(1)HQAFFDK	0.219	3.00E-03	2.899	1.077
mRNA.AJAP09740	K	Lysine-specific demethylase 6A	NKPSFGRVMTK(1)AAR	1.607	1.11E-02	0.923	0.042
mRNA.AJAP16542	K	Melanotransferrin	EHVK(1)SAAK	1.375	3.19E-03	1.224	0.356
mRNA.AJAP18303	K	Methylmalonate-semialdehyde dehydrogenase [acylating], mitochondrial	EIAK(1)NITLEQGK	0.501	1.72E-21	/	/
	K		VQCNMGAK(1)NHGVVMPDANK	0.346	6.37E-26		
mRNA.AJAP22849	K	Methylmalonyl-CoA epimerase, mitochondrial	ATDMYK(1)NVLGAK	0.770	3.03E-05	1.299	0.559
mRNA.AJAP12409	K	Ornithine aminotransferase, mitochondrial	GVPK(1)NQAK	0.825	4.03E-07	0.858	0.060
mRNA.AJAP02385	K	Persulfide dioxygenase ETHE1, mitochondrial	LSK(1)SQEDFIK	0.497	2.52E-04	/	/
mRNA.AJAP05291	K	Probable low-specificity L-threonine aldolase 1	ELADK(1)HGVQIHLDGAR	0.639	9.81E-05	/	/
	K		TVK(1)NLADGSLDLR	0.665	4.95E-04		
mRNA.AJAP18780	K	Protein-glutamine gamma-glutamyltransferase 4	GTLVHAQAVDK(1)FEPNK	1.211	1.28E-12	/	/
mRNA.AJAP04310	K	Protein-glutamine gamma-glutamyltransferase K	SSGNK(1)TSLK	1.234	3.54E-03	0.906	0.333
	K		AALK(1)SEGR	0.738	1.77E-04		
mRNA.AJAP04248	K	Pyrroline-5-carboxylate reductase	ETGK(1)HPGQVK	0.790	7.81E-04	2.360	1.320
mRNA.AJAP22561	K	Serine hydroxymethyltransferase, cytosolic	VLEAASIVCNK(1)NTCPGDK	1.307	1.48E-04	1.111	0.136
mRNA.AJAP18282	K	Serine hydroxymethyltransferase, mitochondrial	EYQFQTVK(1)NSK	0.548	3.98E-05	/	/
	K		NTCPGDK(1)SALVPGGMR	0.506	3.79E-14		
	K		GKDVMYDLEK(1)K	0.378	1.26E-04		
mRNA.AJAP12027	K	Tricarboxylate transport protein, mitochondrial	YK(1)NTMDCIVK	0.484	5.86E-04	0.902	0.332
mRNA.AJAP03833	K	Tryptophan 2,3-dioxygenase	SVMDSLK(1)NVELVK	0.715	6.97E-05	0.707	0.457
	K		IFENTFGLK(1)HK	0.742	4.55E-05		
	K		GVK(1)GTIEDIK	0.796	4.61E-03		
**Coenzyme transport and metabolism**							
mRNA.AJAP07659	K	D-3-phosphoglycerate dehydrogenase	ELGVEVK(1)TTHEDGPR	1.974	9.02E-23	0.835	0.061
mRNA.AJAP17714	K	5-formyltetrahydrofolate cyclo-ligase	GK(1)AYYDTYLK	0.660	1.30E-03	0.448	0.097
**Lipid transport and metabolism**							
mRNA.AJAP20934	K	15-hydroxyprostaglandin dehydrogenase [NAD(+)]	K(1)TLDHFK	0.425	4.88E-03	0.934	0.149
	K		GIDFHK(1)FR	0.448	2.14E-02		
	K		TLDHFK(1)R	0.531	1.52E-02		
mRNA.AJAP24898	K	2,4-dienoyl-CoA reductase, mitochondrial	LDPTGAFSSK(1)AEHR	0.739	3.65E-25	1.230	0.289
mRNA.AJAP14702	K	3-hydroxyisobutyrate dehydrogenase, mitochondrial	QVSRFIK(1)R	0.781	1.56E-02	1.957	0.479
mRNA.AJAP08086	K	3-ketoacyl-CoA thiolase, mitochondrial	GETTMDILK(1)K	0.827	2.72E-04	0.957	0.330
mRNA.AJAP00667	K	Acetyl-CoA acetyltransferase, cytosolic	K(1)NTIEVTTDEFPR	0.689	1.87E-06	0.918	0.132
mRNA.AJAP01146	K	Acetyl-CoA acetyltransferase, mitochondrial	EEQDQFAVSSYK(1)K	1.275	1.23E-06	0.918	0.132
	K		DLK(1)AVFEK	0.402	2.19E-02		
mRNA.AJAP18357	K	Acyl-CoA dehydrogenase family member 10	QFK(1)HGQSNPTYFVGYGGK	0.644	9.56E-08	0.785	0.102
mRNA.AJAP04922	K	Acyl-CoA dehydrogenase family member 9, mitochondrial	QDK(1)ITGFIVER	0.658	5.62E-03	1.089	0.142
mRNA.AJAP11753	K	Acyl-CoA synthetase family member 2, mitochondrial	TIGEALQDSAEK(1)HPNK	0.759	2.99E-07	0.861	0.178
mRNA.AJAP19459	K	Acyl-CoA-binding protein	PGMLDMK(1)GK	1.249	6.58E-03	0.511	0.361
mRNA.AJAP03462	K	Acyl-CoA-binding protein	TLTTK(1)PSDSDMLK	0.575	1.38E-102	0.511	0.361
mRNA.AJAP06802	K	Alcohol dehydrogenase [NADP(+)] A	GNICIPK(1)SVTPSR	0.800	3.05E-07	0.934	0.149
mRNA.AJAP23308	K	Carbonyl reductase [NADPH] 1	FHQLDITK(1)R	1.229	6.73E-04	0.786	0.252
mRNA.AJAP07066	K	Cholinesterase	TFLCSESK(1)FGR	1.235	1.06E-04	0.820	0.112
mRNA.AJAP16068	K	D-2-hydroxyglutarate dehydrogenase, mitochondrial	GGSVSAEHGLGFK(1)K	0.605	1.84E-07	0.673	0.080
mRNA.AJAP24477	K	Dihydropteridine reductase	ALVK(1)NSDLMFK	0.814	9.66E-05	1.161	0.227
mRNA.AJAP30166	K	Enoyl-CoA hydratase, mitochondrial	AVALAEK(1)ISR	0.751	4.72E-05	/	/
	K		VGK(1)NSNVGLIK	0.617	1.27E-03		
mRNA.AJAP23751	K	Hydroxysteroid dehydrogenase-like protein 2	DFIK(1)MFK	0.550	5.91E-03	0.673	0.080
	K		DGANIVIAAK(1)TATPHPK	0.673	9.67E-19		
mRNA.AJAP27168	K	Long-chain specific acyl-CoA dehydrogenase, mitochondrial	AFGK(1)TLSK	0.734	7.35E-03	0.722	0.202
	K		TLSK(1)LQTIQHK	0.475	2.61E-05		
mRNA.AJAP17670	K	Medium-chain specific acyl-CoA dehydrogenase, mitochondrial	IIPK(1)AAHYDK	0.740	4.95E-03	/	/
mRNA.AJAP12788	K	Peroxisomal multifunctional enzyme type 2	AVVTVK(1)PPDRAPDASMSEK	0.793	1.01E-04	/	/
mRNA.AJAP17330	K	Trifunctional enzyme subunit alpha, mitochondrial	GEQHVYK(1)QLDGK	0.642	1.05E-05	0.908	0.105
	K		NSFGK(1)PQK	0.738	1.04E-02		
	K		GCYVYGSGK(1)TR	0.756	1.03E-04		
	K		EVNSEAVEILK(1)K	0.627	1.47E-04		
	K		QLDGK(1)AR	0.549	1.61E-03		
mRNA.AJAP02420	K	Trifunctional enzyme subunit beta, mitochondrial	AK(1)TAGAK	0.579	2.49E-02	0.908	0.105
	K		AMNSDHFAK(1)K	0.752	1.46E-02		
mRNA.AJAP02098	K	Very long-chain specific acyl-CoA dehydrogenase, mitochondrial	GFGGVSSGPPEK(1)K	0.791	4.55E-03	0.625	0.146
mRNA.AJAP16132	K	Vigilin	GAK(1)NCVDGAK	0.709	1.78E-03	0.769	0.087
**Carbohydrate transport and metabolism**							
mRNA.AJAP27703	K	Fructose-bisphosphate aldolase A	CVLK(1)ISEHTPSTLAMK	1.433	6.22E-13	1.184	0.273
	K		EYK(1)AAGADFAK	1.209	2.49E-03		
	K		AAGADFAK(1)WR	1.579	2.98E-12		
mRNA.AJAP16289	K	Probable phosphoglycerate kinase	QDK(1)FSLTPVAK	0.750	2.81E-03	0.784	0.280
mRNA.AJAP16576	K	Phosphoacetylglucosamine mutase	TGVK(1)HLHHK	0.645	7.08E-03	1.308	0.572
	K		VPVACVK(1)TGVK	0.718	2.79E-05		
mRNA.AJAP02728	K	Glucose-6-phosphate isomerase	ASGMAADK(1)IEK	0.742	1.55E-03	1.329	0.669
**Nucleotide transport and metabolism**							
mRNA.AJAP03200	K	Inosine-uridine preferring nucleoside hydrolase	TTWHTK(1)K	1.762	1.04E-04	0.755	0.223
	K		ADVNK(1)FHK	1.784	8.58E-03		
mRNA.AJAP27803	K	UMP-CMP kinase	IVEK(1)FGFK	0.770	7.67E-03	2.147	0.426

### Pan-acetylation western blotting analyses

**Figure 7:**
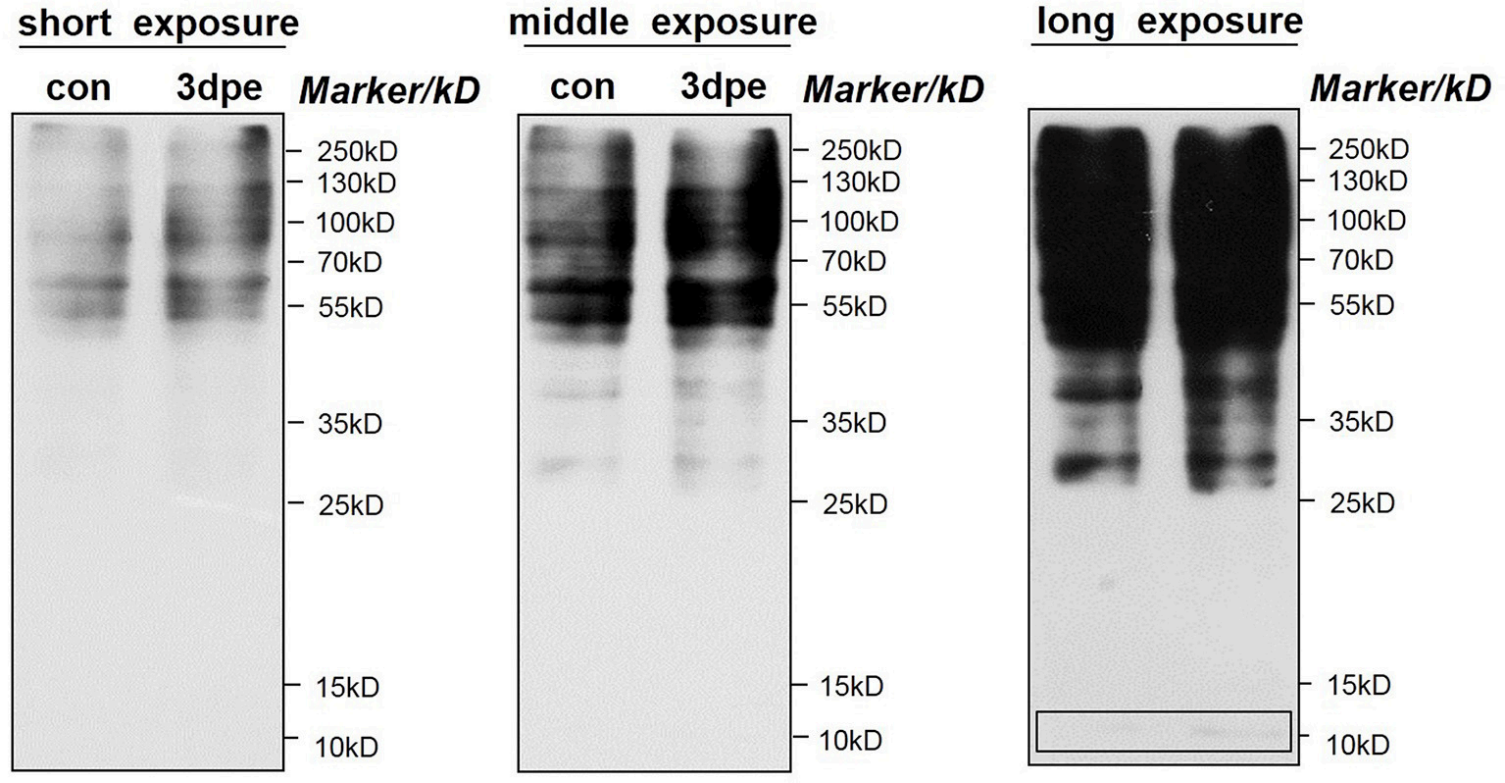
Western blotting of pan-acetylation during intestine regeneration in sea cucumbers. Cell lysate samples contain 20 μg of total protein per lane. Proteins with a molecular weight of ~10 kb are likely histone family member.

The overall acetylation regulation pattern of all proteins was determined by western blotting using a pan anti-acetyllysine antibody. As shown in Figure 7, many proteins were acetylated in both normal and regenerative intestine, but the overall acetylation levels were upregulated during intestine regeneration. Notably, consistent with the acetylated proteomic results, proteins with a molecular weight of ~10 kDa, which were most likely members of the histone family, were upregulated during intestine regeneration. Those findings confirmed that acetylation levels were significantly altered during intestine regeneration, suggesting acetylation probably played an important role in intestine regeneration in sea cucumbers.

## Discussion

PTM can influence protein folding, activity, stability, antigenicity, intracellular localization, and interaction with other proteins or with nucleic acids (Soppa, 2010). To date, more than 200 different PTMs have been described, among which reversible protein phosphorylation and acetylation are prominent and ubiquitous regulatory mechanisms (Van Noort et al., 2012). Herein, we analyzed PTM mechanisms during intestine regeneration in sea cucumbers for the first time, and revealed the importance of protein acetylation-based regulation.

### Cytoskeleton biogenesis

Cytoskeletal proteins play very important roles in cell dedifferentiation and migration during intestine regeneration in sea cucumbers (Murray and García-Arrarás, 2004; Ortiz-Pineda et al., 2009). In this study, phosphorylation and acetylation levels of most cytoskeletal proteins were upregulated, implying that intestine regeneration was regulated through protein phosphorylation (Tables 2, 3, Figure 6). S site hyperphosphorylation (>2-fold upregulation) of the tubulin beta chain-like and neural alfa2 tubulin was detected in regenerative intestine, even though protein expression remained relatively constant during intestine regeneration (Sun et al., 2017c). Acetylation modification of tubulin is known to alter the selectivity of kinesin-1 translocation, and lead to the formation of multiple axons, which is critical for neuronal development and function (Hammond et al., 2010). Interestingly, we found that phosphorylation levels were also upregulated during regeneration, which implied that PTM played an important role in regulating the functions of tubulin. These findings supported the “tubulin-code” hypothesis that predicts different tubulin genes or posttranslational modifications confer variation in the carboxy-terminal tail, resulting in unique interactions with microtubule-associated proteins for specific cellular functions (Sirajuddin et al., 2014).

Filamin-A and filamin-C showed significantly upregulated acetylation at multiple sites during intestine regeneration (Table 3). These actin-binding proteins are crucial in cell adhesion and spreading, which are critical for development, tissue remodeling, and wound healing (Kim H. et al., 2010; Fujita et al., 2012). It has been reported that filamin A was required in injured axons for HDAC5 activity and axon regeneration (Cho et al., 2015). Herein, we inferedthat filamin was modified by acetylation to regulate regeneration in sea cucumbers.

### Protein synthesis and modification

#### Transcription factors, rna processing, and modification

Phosphorylation of CASP-like transcription factor was upregulated 2.454-fold, even though protein expression was not altered (Table 2). This CCAAT displacement protein transcription factor is believed to function in the tethering of transport vesicles and organization of the Golgi stack (Gillingham et al., 2002). Hence, we propose that phosphorylation of CASP-like transcription factor may play an important role in organizing the Golgi stack to regulate protein synthesis. Hyperphosphorylation of pre-mRNA-processing-splicing factor 8 (Prpf8) and neuroblast differentiation-associated protein AHNAK-like was detected in regenerative intestine, and upregulation was 1.724- and 2.257-fold compared with normal intestine (Table 2). Prpf8 is a highly conserved component of both major and U12-dependent minor spliceosomes, and this protein affects transcript splicing, cell survival, and myeloid differentiation in zebrafish (Keightley et al., 2013). AHNAK was found to be the most prominent component of extracellular vesicles, and this protein increases the motility of neighboring fibroblasts (Silva et al., 2016). The present study might suggest phosphorylation activates various factors associated with RNA processing and modification to promote intestine regeneration in sea cucumbers.

It is worth noting that the acetylation level of histone acetyltransferase (HAT) p300 was significantly upregulated at five K sites during intestine regeneration, while protein expression remained constant (Table 3). HATs catalyze histone acetylation which facilitates transcription (Carrozza et al., 2003). HAT p300 acetylates pax5 and strongly enhances pax5-mediated transcriptional activity (He et al., 2011). It has been reported that p300 targets both the epigenome and transcription to unlock a post-injury silent gene expression programme that supports axonal regeneration (Gaub et al., 2011). Hence, acetylation mediated transcription, which played a very important role in intestine regeneration in *A. japonicus*. The underlying mechanisms remain to be uncovered in future research.

#### Translation, ribosomal structure, and biogenesis

Phosphorylation levels of 20 proteins associated with translation, ribosomal structure, and biogenesis were upregulated, and acetylation levels of 18 proteins were altered, suggesting phosphorylation and acetylation played important roles in these aspects of protein synthesis during intestine regeneration (Tables 2, 3). Eukaryotic translation initiation factor 4B-like (eIF4B) and translation initiation factor 2 subunit alpha were hyperphosphorylated 1.757- and 1.513-fold at S and T sites, respectively (Table 2). The phosphorylation state of eukaryotic translation initiation factors positively correlated with both translation and growth rates in the cell (van Gorp et al., 2009). eIF4B, a well-known hyperphosphorylated protein, played a critical role during the initiation of protein synthesis, and its activity could be regulated by multiple phosphorylation events, with phosphorylation of serine essential for optimal translational activity (Duncan and Hershey, 1984; van Gorp et al., 2009). Thus, phosphorylation of translation-related proteins might promote protein synthesis, which is essential for intestine regeneration.

Acetylation levels of 11 ribosomal proteins were altered during intestine regeneration, which implied that acetylation was important for the functions of ribosomal proteins. Three ribosomal proteins, L7, S5, and S18, were acetylated in *Salmonella typhimurium*, which facilitated proton exchange and catalysis (Vetting et al., 2008), and 30 out of 68 ribosomal proteins were found to be N-terminal-acetylated in yeast (Arnold et al., 1999). Thus, acetylation regulated the function of ribosomal proteins, which facilitated intestine regeneration in sea cucumbers.

#### Posttranslational modification, protein turnover, chaperones

In this category, four proteins including E3 ubiquitin-protein ligase UBR4 etc. were found to be hyperphosphorylated during intestine regeneration, while protein expression levels remained constant (Table 2). Furthermore, 15 proteins, including [F-actin]-methionine sulfoxide oxidase MICAL2 etc., were differentially acetylated during intestine regeneration in *A. japonicus* (Table 3). Hence, acetylation appeared to regulate their functions more dramatically than phosphorylation during intestine regeneration. Acetylation levels of maleylacetoacetate isomerase, peptidyl-prolyl cis-trans isomerase, and protein disulphide-isomerase were significantly changed. We therefore speculated that the activity of the isomerase family members may be altered via acetylation and deacetylation, which might play a role in regeneration in *A. japonicus*. Similar results have been reported previously, including the role of Cyclophilin A in immunity and viral infection (Lammers et al., 2010), prolyl isomerase Pin1 in orchestrating p53 acetylation (Mantovani et al., 2007), and prolyl isomerase Pin1 in fibroblast growth factor 2-induced osteoblast differentiation (Yoon et al., 2014).

#### Chromatin structure and dynamics

Chromatin structure and dynamics have a major impact on all nuclear processes (Eberharter and Becker, 2002). Nine proteins related to this category were differentially acetylated or deacetylated (Table 3). Acetylation may be more vital to the regulation of chromatin structure and dynamics than phosphorylation. Among all regulated proteins, eight belonged to the histone family. Histone acetylation is the mostdocumented form of protein acetylation, and is known to direct histone assembly and help regulate the unfolding and activity of genes (Grunstein, 1997). Gene expression is affected by the positioning of nucleosomes relative to regulatory sequence elements, which are regulated by site-specific acetylation of nucleosomal histones (Eberharter and Becker, 2002). Histone acetylation can influence all three aspects of chromatin organization, which is central to the switch between permissive and repressive chromatin structure (Eberharter and Becker, 2002). In this study, we also concluded that acetylation modification might play a very important role in regulating gene expression to facilitate intestine regeneration in sea cucumbers.

### Signal recognition and transduction

Rapid and accurate transmission of signals from cell surface receptors to the nucleus is clearly dependent on protein phosphorylation (Karin and Hunter, 1995), which is also verified by our present results t. Phosphorylation levels of 18 proteins were significantly altered (Table 2, Figure 6). From these results, we concludedthat protein phosphorylation might play a very important role in signal recognition and transduction during intestine regeneration. The cGMP-dependent protein kinase (PKG) was dramatically hyperphosphorylated by over 2.483- to 4.720-fold, during intestine regeneration. It has been demonstrated that phosphorylation plays a central role in regulating the activation and signaling lifetime of protein kinases (Newton, 2003). We also observed the phosphorylation levels of four sites in titin-like protein were downregulated, while the protein expression level was unaltered (Table 2). The role of titin phosphorylation in the muscle signaling mechanisms is well-documented (Tskhovrebova and Trinick, 2003), and evidence has suggested that both the Z-line and M-line ends of the molecule are components of signaling pathways that control tension- and protein-turnover-related processes (Tskhovrebova and Trinick, 2003).

### Energy production and conversion

In contrast to the “signal recognition and transduction” category, almost all modified proteins related to the “energy production and conversion” term were regulated by deacetylation (Table 3, Figure 6). The results showed that 27 proteins were hypoacetylated, but only three proteins were hyperacetylated. Two points are noteworthy in this study; (1) acetylation levels of most proteins were downregulated, and (2) a large number of the modified proteins were related to ATP/ADP production and conversion. During the early stages of intestine regeneration, *A. japonicus* stops feeding due to loss of intestines after evisceration, hence an energy management strategy is vital to the smooth progress of regenerative processes. It has been established that reversible acetylation-deacetylation of PGC-1α, an important regulator of energy homeostasis, is related to energy sensors that regulate mitochondrial energy homeostasis to maintain appropriate energy levels (Jeninga et al., 2010). In our study, almost all proteins were hypoacetylated, which may be a wise way to save energy. ATP synthase family members including mitochondrial ATP synthase subunit alpha (d, f, g, and o), were significantly hypoacetylated, by 0.495- to 0.796-fold. ATP synthase, an evolutionarily conserved protein complex, uses chemiosmotic energy stored as a gradient across the mitochondrial inner membrane to convert ADP and orthophosphate to ATP (Vassilopoulos et al., 2014). A previous study verified that SIRT3 deacetylates ATP synthase F1 in response to nutrient- and exercise-induced stress (Vassilopoulos et al., 2014). For *A. japonicus*, intestine regeneration is a type of nutrient- or stimulation-related stress. Hence, deacetylation of proteins related to energy production and conversion may manipulate energy distribution in response to changes in nutrient and energy stress to ensure continuation of intestine regeneration processes.

### Substance transport and metabolism

Substance metabolism must be affected by energy distribution and hypometabolism during intestine regeneration. From the results, we predicted that reversible acetylation of substance transport and metabolism-related proteins in regenerative intestine might be more vital than reversible phosphorylation, and regulation of reversible acetylation appeared mainly to involve amino acid and lipid transport and metabolism. Many amino acid transferases, including aminomethyl transferase, aspartate aminotransferase, and ornithine aminotransferase, were hyper- or hypoacetylated, implying that reversible acetylation altered the three-dimensional structure and enzymatic functions to regulate amino acid transport to support intestine regeneration. During the early stages of intestine regeneration, key nutrient substances must be transported in a timely manner to regenerative blastema. In our previous study, we found that over half of proteins related to amino acid transport and metabolism were upregulated (Sun et al., 2017c). Taken together, these results suggested that amino acid related to transport and metabolism were rapidly manipulated during blastema formation in the early stages of intestine regeneration in *A. japonicus*. Additionally, acetylation levels of almost all proteins related to lipid transport and metabolism were downregulated during intestine regeneration, which is comparable to our previous study showing that expression of all proteins related to this term were significantly downregulated at 3 dpe (Sun et al., 2017c). Hence, we speculated that lipid metabolism could be postponed to provide extra energy for the intermediate or later stages of regeneration. This finding might indicate that sea cucumbers could adopt appropriate energy and metabolism strategies to guarantee the smooth progress of regeneration.

## Conclusions

This study provided a global view of the role of protein phosphorylation and acetylation in intestine regeneration in sea cucumbers. To the best of our knowledge, it is the first such phospho- and acetylproteomics study in sea cucumbers. We found that the identified differentially modified proteins regulated regeneration from five different aspects, and phosphorylation and acetylation played different roles. These results not only improved our understanding of the regulatory mechanisms of intestine regeneration, but also enhanced our understanding of phosphorylation- and acetylation-based regulation in sea cucumbers even echinoderm.

## Ethics statement

This study was carried out in accordance with the recommendations of Welfare ethics of experimental animals and safety inspection system of animal experiments, laboratory animal management and ethics Committee of IOCAS. The protocol was approved by the laboratory animal management and ethics Committee of IOCAS.

## Author contributions

LS and HY conceived the paper. LS, CL, XL, and HY designed the experiments and analyzed the data. LS wrote the paper. LX, DH, JS and LZ revised the paper.

### Conflict of interest statement

The authors declare that the research was conducted in the absence of any commercial or financial relationships that could be construed as a potential conflict of interest.

## Supplementary material

Data Sheet 1 All phosphorylated proteins during intestine regeneration in sea cucumbers.

Data Sheet 2 All acetylated proteins during intestine regeneration in sea cucumbers.
